# Tiny Carriers, Big Impact: How *Tannerella forsythia* Extracellular Vesicles Drive Oral Diseases, and Beyond

**DOI:** 10.3390/ijms27219880

**Published:** 2026-11-04

**Authors:** Zhixian Shen, Guojun Xu, Yongjie Li, Hengxiang Liu, Zishuai Chen, Shengxiang Chen, Qinglang Han, Zifan Wang, Hai He

**Affiliations:** 1 Key Laboratory of Biotechnology and Bioengineering of State Ethnic Affairs Commission, Biomedical Research Center, School of Bioengineering, Northwest Minzu University, Lanzhou 730030, China; szx1658@outlook.com (Z.S.); guojunxu10@outlook.com (G.X.); yongjieli2026@outlook.com (Y.L.); 18324841356@163.com (H.L.); czs2410188708@outlook.com (Z.C.); zxc18875818081@outlook.com (S.C.); qinglanghan@outlook.com (Q.H.); 2 Gansu Animal Cell Technology Innovation Center, Northwest Minzu University, Lanzhou 730030, China; 3 Key Laboratory of Oral Diseases of Gansu Province, School of Stomatology, Northwest Minzu University, No. 1 Xibeixincun Baiyin Road, Chengguan District, Lanzhou 730030, China; 4 Key Laboratory of Stomatology of State Ethnic Affairs Commission, School of Stomatology, Northwest Minzu University, No. 1 Xibeixincun Baiyin Road, Chengguan District, Lanzhou 730030, China

**Keywords:** anti-virulence therapy, extracellular vesicles, inflammasome, outer membrane vesicles, periodontitis, S-layer, *Tannerella forsythia*, Th1 polarization, type IX secretion system

## Abstract

Periodontitis influences systemic health through mechanisms that extend far beyond the periodontal pocket, yet the vehicles that mediate this dissemination remain incompletely defined. Within the “red complex” of periodontitis, *Tannerella forsythia* (Tf) has long been overshadowed by *Porphyromonas gingivalis* and *Treponema denticola*, and its extracellular vesicles (EVs) have been correspondingly neglected. This review synthesizes a decade of evidence to reposition Tf EVs as a distinct nanoscale virulence-delivery platform. Their biogenesis couples outer-membrane budding to the type IX secretion system (T9SS), and the crystalline surface layer (S-layer) appears to be actively reprogrammed—rather than passively shed—to accommodate vesicle curvature, a mechanism so far supported by compositional evidence. Their cargo is selectively enriched in T9SS substrates, TonB-dependent receptor/SusD-like nutrient-acquisition proteins, and extensively O-glycosylated virulence factors. Mechanistically, as demonstrated directly at the vesicle level in cellular models, Tf EVs engage Toll-like receptor 2 (TLR2), autonomously prime and activate the inflammasome, and drive IL-12-dependent Th1 polarization. These properties define a “purely pro-inflammatory” immunological identity that distinguishes Tf EVs from those of *P. gingivalis* and *Fusobacterium nucleatum*. Signature cargo, including BspA, miropin, and PepO, suggests potential links between Tf EVs and cardiovascular, articular, and pulmonary diseases; however, these systemic associations are currently inferred mainly from whole-cell, isolated-virulence-factor, or epidemiological evidence, rather than from direct Tf EV-level experiments, and are therefore presented here as testable hypotheses. We critically appraise isolation and standardization practices, delineate priority gaps such as unverified vesicle subtypes and unexplored small RNA cargo, and propose a four-step roadmap from subtype isolation to clinical cohorts. Tf EVs warrant recognition as an independent research object and a candidate druggable target for periodontitis, whereas their contribution to its systemic comorbidities awaits direct causal validation at the EV level.

## 1. Introduction

Periodontal disease progresses from reversible gingivitis to irreversible destruction of the tooth-supporting tissues [1], a trajectory driven by subgingival biofilm dysbiosis that underlies periodontal pocket formation, attachment loss, and alveolar bone resorption [2]. At the terminal stage of this ecological succession, the Gram-negative obligate anaerobe *Tannerella forsythia* (Tf), together with *Porphyromonas gingivalis* (Pg) and *Treponema denticola* (Td), constitute the “red complex” of periodontitis—a consortium of niche-linked late colonizers that cooperatively remodel the periodontal pocket microenvironment [3,4], whose detection levels correlate strongly with the severity of chronic periodontitis [5,6]. The burden is formidable: severe periodontitis affects 11.2% of the global population, ranks as the sixth most prevalent disease of humankind, and is epidemiologically associated with diabetes, cardiovascular disease (CVD), cancer, and dementia [1]. Yet within the red complex, Tf has long been overshadowed by Pg and Td; both the characterization of its virulence determinants and the mechanisms underlying its host interactions remain conspicuously underexplored, and few dedicated reviews in recent years have systematically catalogued its virulence repertoire [7]. Critically, Tf has also been linked to conditions beyond the periodontium, although mainly at the epidemiological level: infection with—and serological exposure to—this organism is associated with elevated CVD risk [8], linked to arthritis, and closely associated with periodontitis in patients with obesity or type 2 diabetes mellitus (T2DM) [9]. At the level of an individual virulence factor, its surface adhesin BspA has been shown to drive macrophage foam-cell formation and to accelerate atherosclerotic plaque progression in ApoE-/- mice [7,10]; because these data were obtained with whole bacterial cells and a single recombinant adhesin, rather than with EVs, they support a potential “periodontal pathogen–atherosclerosis” axis, whose vesicle-mediated contribution remains to be established. Collectively, these findings indicate that Tf virulence factors possess pathogenic potential far beyond the periodontal pocket; however, the mechanisms by which they contribute to systemic disease remain poorly defined.

**Figure 1:**
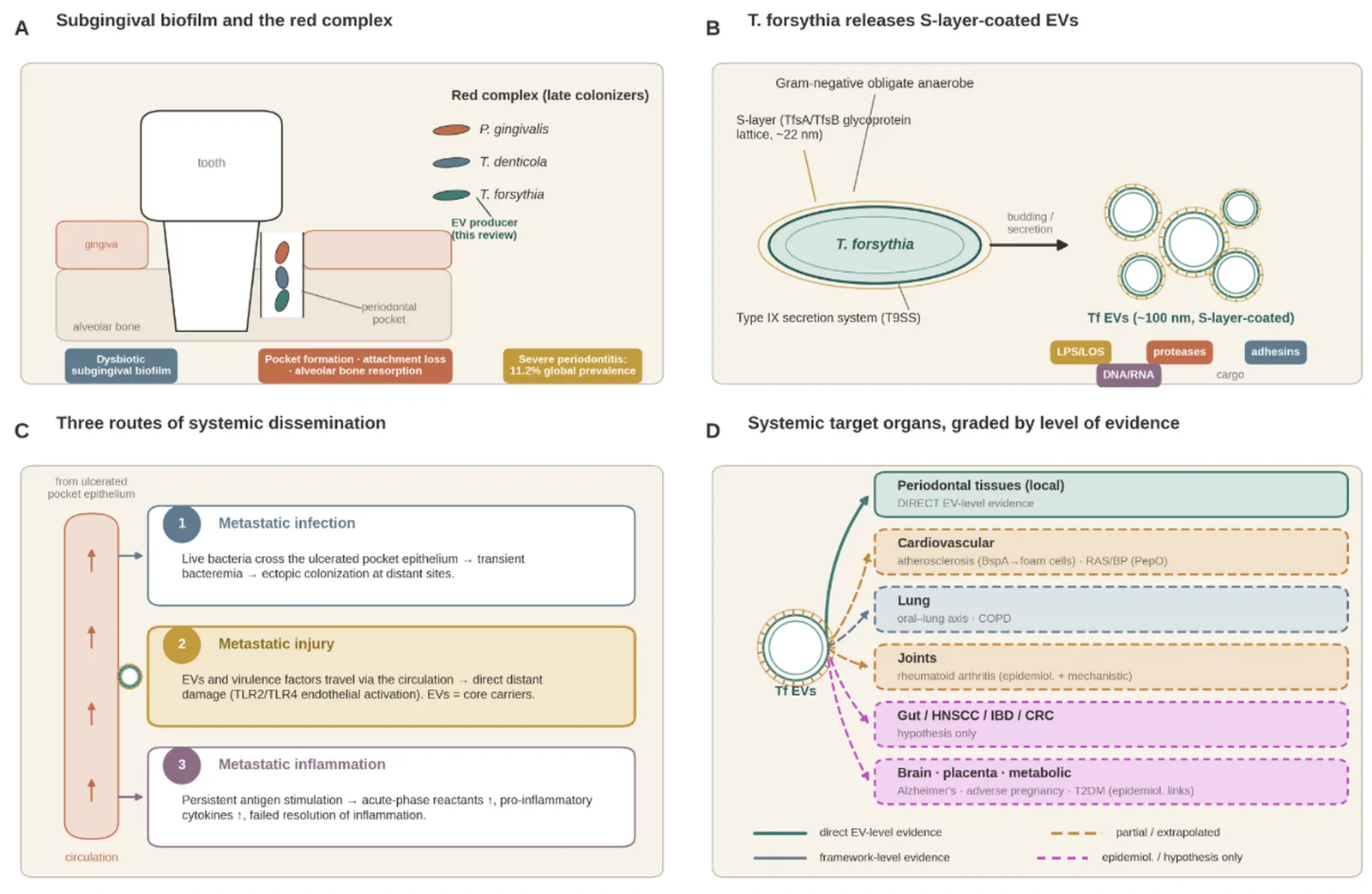
Overview of *Tannerella forsythia* (Tf) EVs and their systemic dissemination. (**A**) Tf within the “red complex” and the periodontal pocket. (**B**) S-layer-coated Tf EVs (~100 nm) and their cargo. (**C**) Three dissemination routes, with EV-mediated metastatic injury as the core EV route (red arrows indicate transit through the circulation). (**D**) Systemic target organs, graded by level of evidence (arrow line styles denote evidence grades). Abbreviations: EV, extracellular vesicle; LPS, lipopolysaccharide; LOS, lipo-oligosaccharide; TLR, Toll-like receptor; RAS, renin–angiotensin system; BP, blood pressure; COPD, chronic obstructive pulmonary disease; HNSCC, head and neck squamous-cell carcinoma; IBD, inflammatory bowel disease; CRC, colorectal cancer; T2DM, type 2 diabetes mellitus.

This observation raises a central question: how does Tf virulence reach distant organs? A recently proposed tripartite dissemination framework deconstructs the oral–systemic connection into three routes [11]: (i) metastatic infection, in which viable bacteria breach the ulcerated pocket epithelium, enter the circulation to produce transient bacteremia, and colonize ectopic sites; (ii) metastatic injury, in which virulence factors and metabolites disseminate hematogenously to inflict direct remote damage—exemplified by TLR2/TLR4-mediated activation of vascular endothelium and the ensuing endothelial dysfunction; and (iii) metastatic inflammation, in which persistent stimulation by periodontal antigens provokes systemic inflammation, manifested by upregulated acute-phase reactants, elevated pro-inflammatory cytokines, and failed resolution of inflammation [11]. Within this framework, bacterial extracellular vesicles (EVs; termed outer-membrane vesicles, OMVs, in Gram-negative bacteria) have emerged as the principal vehicles of metastatic injury, encapsulating and delivering virulence cargoes—lipopolysaccharide, proteases, and adhesins—to distant organs without requiring colonization by viable bacteria [11,12]. This route has gained preliminary clinical support at the whole-organism level: red-complex members, including Tf, have been detected in airway samples from patients with chronic obstructive pulmonary disease (COPD) [11,13]. Whether Tf EVs themselves reach the lung has not been demonstrated; however, the pulmonary capillary bed—uniquely exposed to bacteremia and circulating OMVs originating from diseased periodontal tissues—is a plausible site at which circulating EVs could drive local endothelial activation and a metastatic inflammatory cascade [11,13]. Notably, the concept that OMVs from oral pathogens disseminate via the circulation to drive distant disease has been systematically articulated for periodontal pathogens such as Pg: Catalan et al. demonstrated that oral pathogens mediate remote systemic effects by releasing OMVs capable of traversing epithelial and endothelial barriers into the circulation [14], and the contribution of this mechanism to periodontitis-associated systemic diseases has been extensively reviewed [15,16]. Nevertheless, the protagonists of this narrative have invariably been Pg and *Fusobacterium nucleatum* (Fn); even in reviews dedicated to red-complex membrane vesicles, Tf receives only cursory coverage and limited mechanistic depth [6]. The central thesis of this review is that, despite this long-standing neglect, Tf EVs—distinguished by their S-layer coating, type IX secretion system (T9SS)-coupled release, and unique cargo composition—may prove no less consequential than those of Pg or Fn in the oral–systemic disease axis (Figure 1). To avoid overstating the current evidence, we explicitly distinguish throughout this review between three evidence tiers: effects demonstrated directly with Tf EVs, activities of individual virulence factors or whole bacterial cells, and pathogenic mechanisms proposed by extrapolation.

To substantiate this thesis, we first clarify the EV concept and retrace the research trajectory of Tf EVs. Bacterial EVs are nanoscale lipid-bilayer vesicles shed from the envelope during growth; enriched in virulence factors and readily internalized by host cells, they function as a bona fide “long-range virulence delivery system” [14]. Although Tf EV research began relatively late, the past decade has seen it coalesce into a coherent evidence chain. In 2015, Friedrich et al. provided the first systematic characterization of Tf OMVs, demonstrating that they arise through outer-membrane budding, measure approximately 100 nm in diameter, retain a 22 nm S-layer on their surface, and harbor 175 high-confidence protein cargoes [17]. In 2016, Cecil et al. established their physicochemical fingerprint and a standardized quantification protocol [18]. Subsequent proteomic and O-glycoproteomic analyses revealed enrichment for T9SS substrates and complex glycosylation modifications [19,20]. Mechanistic immunological studies showed that Tf OMVs activate the macrophage inflammasome [21] and, via dendritic cells, induce IL-12-dependent Th1 polarization—a response profile distinct from that elicited by Pg or Td [22]. In 2023, An et al. demonstrated that a quorum-sensing inhibitor attenuates their pro-inflammatory activity [23]. Most recently, in 2025, Waligórska et al. demonstrated that the metalloprotease PepO is released via OMVs and hydrolyzes angiotensin I in vitro, and proposed that Tf EVs could thereby participate in regulation of the renin–angiotensin system—a potential extension of Tf EV function into cardiovascular endocrinology whose in vivo relevance remains to be tested [24]. Individually incremental, yet collectively convergent, these advances now provide a sufficiently mature foundation for the systematic synthesis undertaken in this review.

## 2. The Terminology of EVs

**Figure 2:**
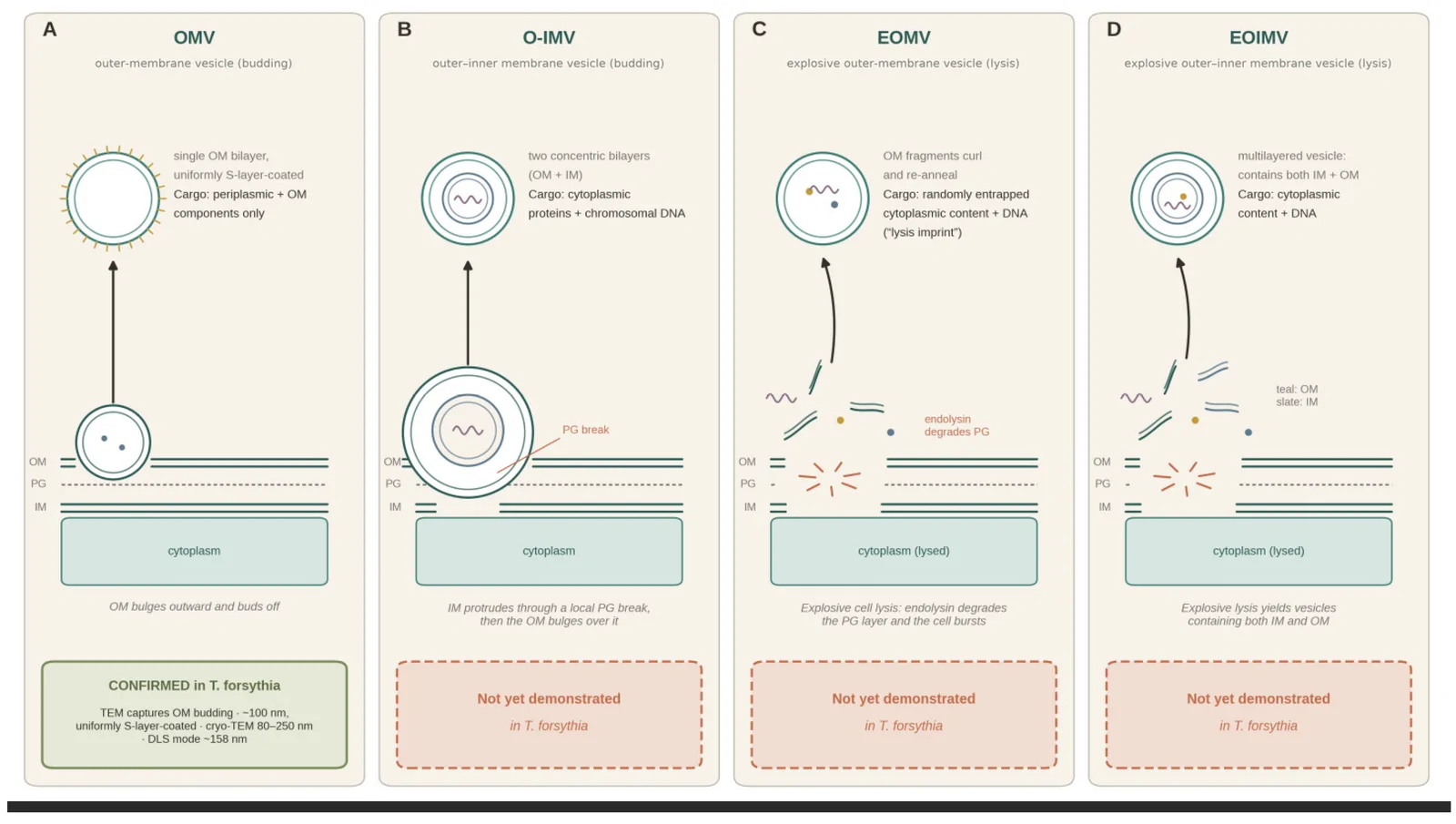
Biogenesis of the four Gram-negative EV subtypes and their status in *T. forsythia*. (**A**) Classical OMVs bud from the outer membrane—the only subtype proven in Tf. (**B**) O-IMVs carry concentric inner- and outer-membrane bilayers, packaging cytoplasmic proteins and DNA. (**C**,**D**) EOMVs and EOIMVs arise from explosive lysis; neither is demonstrated in Tf.

Nanoscale lipid vesicles released by Gram-negative bacteria were collectively designated outer-membrane vesicles (OMVs) in early studies [25]; however, this name carries an implicit presumption of outer-membrane budding and no longer suffices to encompass all bacterially derived vesicles. Based on membrane composition and biogenesis route, Gram-negative EVs can be classified into four subtypes (Figure 2): (i) classical OMVs, formed by localized outward bulging and budding of the outer membrane and containing only outer-membrane and periplasmic constituents; (ii) outer–inner membrane vesicles (O-IMVs), which carry concentric inner- and outer-membrane bilayers whose innermost lumen communicates with the cytoplasm and can therefore package cytoplasmic proteins and chromosomal DNA; and (iii) explosive outer-membrane vesicles (EOMVs) [26] and (iv) explosive outer–inner membrane vesicles (EOIMVs) [27], which arise from the recircularization of membrane fragments after explosive cell lysis, and whose cargo bears a “lysis signature” of randomly entrapped cytoplasmic material. Subtype assignment is not a morphological formality [26]: the biogenesis route directly determines the cargo spectrum, and accurate subtype discrimination is a prerequisite for interpreting EV cargo heterogeneity and inferring biogenetic mechanisms. Accordingly, in the absence of subtype-specific isolation or direct morphological evidence, labeling vesicles as “OMVs” presupposes a budding origin and carries the risk of mechanistic misattribution [26]—if lysis-derived vesicles contaminate a preparation, their cytoplasmic proteins and DNA [28] will be erroneously ascribed to “active sorting” by budding OMVs. Vesicle studies of red-complex bacteria have largely retained the “OMV” designation [6], and the Tf field is no exception [17]; yet this usage rests primarily on morphological observation of budding and physicochemical characterization, rather than on systematic discrimination of the four subtypes, and current preparations are, in fact, mixed populations obtained by differential ultracentrifugation or density-gradient centrifugation [18], from which lysis-derived vesicles have not been excluded. We therefore adopt the umbrella term “Tf EVs” as the default designation throughout this review, reserving “OMV” for contexts in which the subtype has been directly demonstrated. A note on taxonomic scope: the genus *Tannerella* comprises at least two recognized species, *T. forsythia* and *T. serpentiformis*, each encompassing multiple strains, and the “red complex”, although classically defined by the three key species Pg, Tf, and Td, functions within a broader polymicrobial consortium; accordingly, expressions such as “cross-species” are used in this review strictly for comparisons between named species, and strain-level resolution is addressed in Section 4.3.

To date, the only vesicle type directly demonstrated in Tf is the classical budding OMV: transmission electron microscopy (TEM) has directly captured the process of outer-membrane budding, revealing vesicles of uniform size with a mean diameter of approximately 100 nm, the surfaces of nascent vesicles being fully covered by an approximately 22 nm thick S-layer that retains square lattice symmetry [17]. After OptiPrep density-gradient purification, cryo-TEM revealed a vesicle size distribution of 80–250 nm, while dynamic light scattering (DLS) measured fully hydrated diameters of 59–397 nm with a mode of approximately 158 nm [18]. Tf is the only red-complex member coated by an S-layer (the TfsA/TfsB glycoprotein layer, also termed the EDSL) [17]; ultrathin-section TEM confirmed that this structure is preserved intact during vesicle budding [17], distinguishing Tf vesicles from canonical Gram-negative OMVs whose surfaces present a bare LPS outer leaflet. However, no direct evidence yet exists for O-IMVs, EOMVs, or EOIMVs in Tf. Notably, in the fellow red-complex member Pg, a substantial proportion of OMV-associated DNA is resistant to DNase digestion, suggesting a luminal localization [18]; given the similarity between Tf and Pg in OMV biogenesis routes and packaging capacity, DNA within Tf vesicles may, likewise, be internally encapsulated. By subtype definition, luminal DNA is consistent with double-membrane subtypes or lysis-derived vesicles, whereas the luminal cargo of classical budding OMVs is, in principle, periplasm-derived [26]; this finding, therefore, suggests that preparations may contain double-membrane subtypes, but nucleic-acid cargo alone is insufficient for subtype assignment, and verification will require cryo-electron tomography (cryo-ET) to resolve membrane-layer number, together with subtype-specific isolation. Given this state of evidence, this review uniformly employs “Tf EVs” hereafter; when citing primary literature, we retain the original authors’ “OMV” usage, for fidelity to their wording.

## 3. EV Biogenesis and Regulatory Factors

### 3.1. Biogenesis Mechanisms of the EV Subtypes (With Emphasis on the Proven Budding OMV)

#### 3.1.1. The Outer-Membrane Budding Model and S-Layer Adaptation to High Curvature

**Figure 3:**
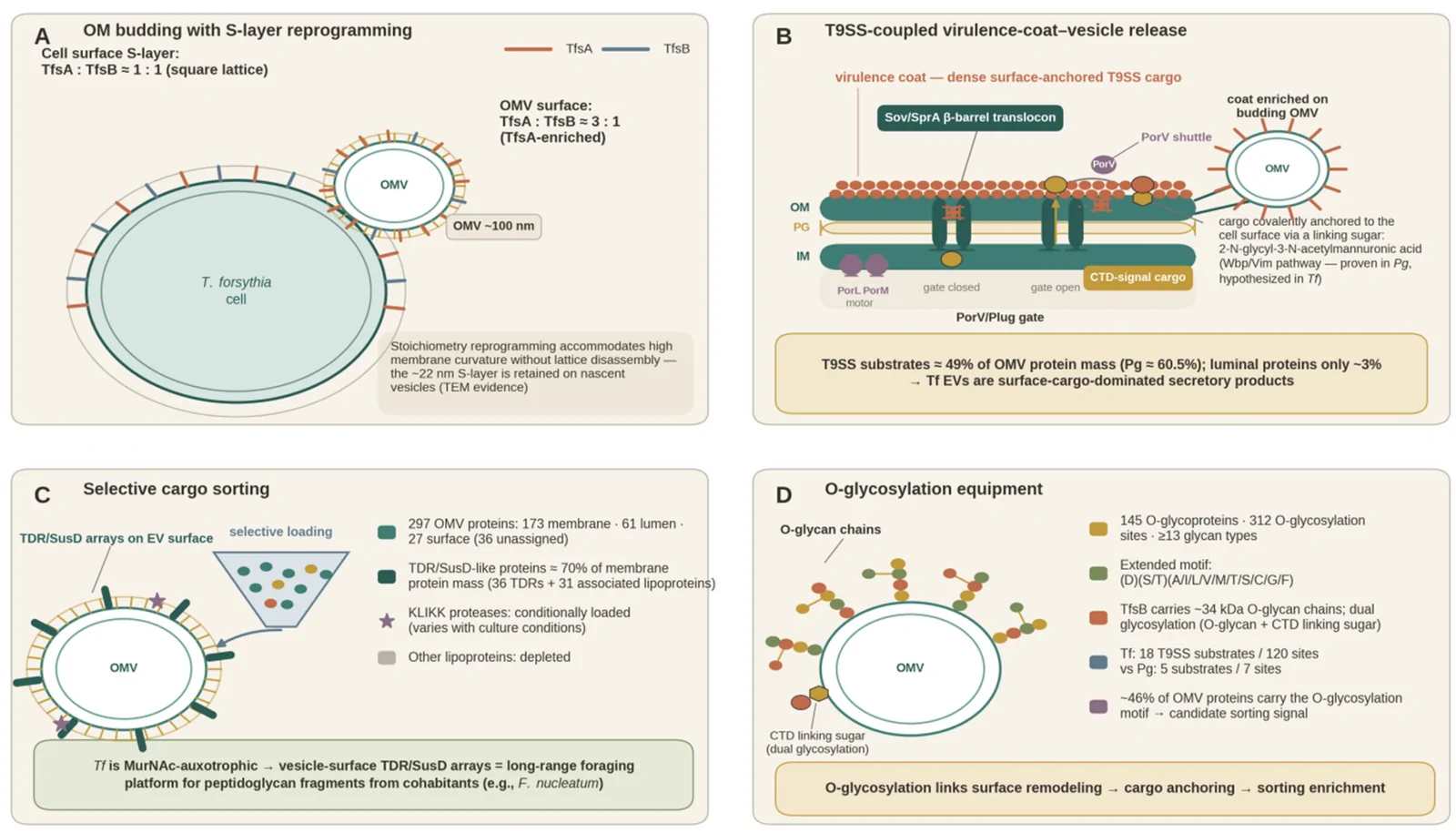
Biogenesis of Tf EVs. (**A**) OM budding with proposed S-layer reprogramming: the TfsA:TfsB ratio shifts from ~1:1 on the cell to ~3:1 on OMVs—interpreted as accommodating high curvature while retaining the ~22 nm lattice; direct experimental confirmation is pending. (**B**) T9SS-coupled virulence-coat release (linking sugar: Pg-proven, Tf-hypothesized); T9SS substrates ≈ 49% of OMV protein mass. (**C**) Selective cargo sorting: TDR/SusD ≈ 70% of membrane mass—a foraging platform for this MurNAc auxotroph. (**D**) Extensive O-glycosylation (TfsB carries ~34 kDa O-glycans)—a candidate sorting signal.

Friedrich et al. used TEM to image *Tannerella forsythia* cells and captured intermediates of outward outer-membrane bulging and budding that give rise to nascent OMVs (Figure 3A); the nascent vesicles had a mean diameter of approximately 100 nm, and their surfaces fully retained the 22 nm thick S-layer glycoprotein shell [17]. This morphological evidence supports biogenesis of Tf vesicles via the classical outer-membrane budding route, although the authors noted that the presence of trace amounts of O-IMV subtypes in the preparations could not be entirely excluded. Unlike most Gram-negative bacteria, the Tf surface is covered by a rigid two-dimensional lattice assembled from TfsA (Tanf_03370) and TfsB (Tanf_03375) in an approximately 1:1 equimolar ratio, whose square arrangement has been resolved by AFM/TEM; deletion of either *tfsA* or *tfsB* disrupts the ordered two-dimensional crystalline lattice, yet an aperiodic proteinaceous layer remains on the outer-membrane surface, and only the double knockout abolishes this outer layer entirely [29]. The critical observation is that the TfsA:TfsB ratio on OMVs shifts to approximately 3:1—that is, TfsA is relatively enriched [19]. Because the native 1:1 regular array cannot be projected geometrically onto a highly curved spherical surface, the authors proposed that Tf actively remodels this stoichiometry to endow the lattice with the conformational freedom needed to accommodate curvature, suggesting that vesiculation is achieved through compositional remodeling, rather than lattice disassembly [19]. Thus, the S-layer has been interpreted not as a passive obstacle to budding, but as being “reprogrammed” into a structural participant in vesicle formation; it must be emphasized, however, that this “active reorganization” is an interpretation inferred from the altered TfsA:TfsB stoichiometry, rather than a directly confirmed process, and its direct demonstration—for example, by real-time imaging of S-layer dynamics during budding—remains outstanding.

#### 3.1.2. The T9SS-Coupled “Virulence Coat–Vesicle Release” Axis

The type IX secretion system (T9SS), unique to the phylum Bacteroidota, is the key to deciphering the cargo composition of Tf OMVs; its machinery encompasses inner-membrane components (PorL/PorM, among others) and outer-membrane-associated components (PorV, Sov/SprA, among others) [30]. Genome-wide essentiality analyses indicate that most T9SS machinery components are essential for secretory function, whereas the majority of substrate cargo proteins are not [31], indicating that the secretion machinery itself is under strong selective constraint. Cross-species cryo-EM evidence (from the *Flavobacterium johnsoniae* model) demonstrates that the giant outer-membrane β-barrel translocon Sov/SprA adopts open and closed gating states through alternating association with PorV or the Plug, driving substrate export across the outer membrane [32]. Substrates bearing a C-terminal domain (CTD) signal are then fixed to the cell surface via covalent “linking sugars”, through the PorV shuttle protein and anchoring complexes [33], forming a dense “virulence coat” that is enriched and released with OMV budding—rendering OMVs a functional extension of the T9SS. Tf possesses a complete set of T9SS orthologs [34]; its specific linking sugar, 2-N-glycyl-3-N-acetylmannuronic acid [33], is proposed to be synthesized via the Wbp/Vim pathway—a hypothetical model extrapolated from genetic experiments in the neighboring species Pg on the basis of homologous gene comparison, which posits that the corresponding sugar in Tf is likewise synthesized, via the Wbp/Vim pathway [20,33,35]—and deletion of its biosynthetic genes in Pg abolishes surface anchoring, directly demonstrating that the linking sugar is the chemical basis of virulence-coat assembly. The quantitative weight of this axis is highly consistent across species: proteomic quantification of Pg OMVs from chemostat cultures showed that T9SS-secreted CTD cargo proteins account for approximately 60.5% of total OMV protein molar abundance [36]—an overwhelmingly dominant share—and approximately 49% in Tf, whereas luminal proteins account for only approximately 3% of molar abundance [19]. This suggests that the “virulence coat–vesicle release” axis may represent a shared framework for EV biogenesis across the red complex, and that Tf EVs are, in essence, secretory products dominated by outer-membrane-surface T9SS cargo.

#### 3.1.3. Proteomic Evidence for Selective Cargo Sorting

Tf EV cargo is not a random sampling of outer-membrane proteins, but the outcome of selective enrichment [37]. Using differential ultracentrifugation combined with LC-MS/MS-based subcellular-localization analysis, Veith et al. identified 872 proteins, of which 297 were assigned to OMVs (173 membrane, 61 luminal, and 27 surface) [19]. The sorting pattern is striking: T9SS substrates are broadly enriched, and 70% (by protein mass) of vesicle membrane proteins are TonB-dependent receptors (TDRs) and their associated SusD-like lipoproteins (36 TDRs and 31 associated lipoproteins, in total) [19]. Given that SusD captures ligands at the surface [38] while TDRs accomplish transmembrane uptake energized by TonB, this overwhelming enrichment suggests that Tf EVs additionally function as a “ligand-capture/nutrient-acquisition platform” [19]—highly consistent with the metabolic background of Tf as an *N*-acetylmuramic acid (MurNAc) auxotroph that must scavenge peptidoglycan fragments from the cell-wall turnover products of commensals such as *Fusobacterium nucleatum* [39]; the dense TDR/SusD arrays on the vesicle surface provide precisely the structural vehicle for such “long-range foraging” [19]. Conversely, other lipoproteins are markedly depleted, further corroborating the selectivity of sorting [19]. In addition, KLIKK proteases are conditionally loaded, their abundance fluctuating with culture conditions—the first indication that the Tf EV cargo spectrum is a secretory output dynamically regulated by environmental signals.

#### 3.1.4. The Role of the O-Glycosylation System in Vesicle Formation and Cargo Loading

Tf possesses one of the most complex protein O-glycosylation apparatuses known in Bacteroidota [40,41]: 145 glycoproteins and 312 O-glycosylation sites have been identified, the motif has been expanded to (D)(S/T)(A/I/L/V/M/T/S/C/G), and at least 13 O-glycan glycoforms are present [20]; the S-layer protein TfsB, alone, bears approximately 34 kDa of glycan chains per polypeptide [20,33], and T9SS substrates generally exhibit a dual glycosylation pattern of “O-glycan modification plus CTD linking-sugar anchoring” [20,33,42]. Comparative analysis shows that the 18 T9SS substrates of Tf, collectively, carry 120 O-glycosylation sites—far exceeding the five substrates and seven sites of Pg [20,43]—and that more than 95% of OMV proteins in both organisms are outer-membrane/surface proteins [19,36,44], suggesting that glycosylation “density” is a distinctive assembly strategy of Tf. The very first Tf OMV study already noted that approximately 46% of OMV proteins contain the O-glycosylation motif [17], hinting that it may act as a sorting signal participating in directed cargo loading. Given that S-layer glycoproteins are, simultaneously, structural components and T9SS-anchored cargo [34], the O-glycosylation system, in fact, spans three processes—“vesicle-surface remodeling, cargo anchoring, and sorting enrichment”—and represents an underappreciated regulatory layer in Tf EV biogenesis.

#### 3.1.5. Knowledge Gap: The Biogenesis of the Lytic Route (EOMV/EOIMV) Remains Unexplored

It must be frankly acknowledged that current knowledge of Tf EV biogenesis rests almost entirely on the budding-OMV model. At the molecular level, Tf lacks studies of peptidoglycan–outer-membrane linkage (Braun’s lipoprotein, OMPLA, and the like) and of membrane-curvature regulation, and, whether the explosive-lysis route (which generates EOMVs/EOIMVs) exists at all, is completely unknown. Given that vesicle-associated DNA has been detected in Tf OMVs [18], part of which is presumed to be DNase-insensitive, owing to a luminal location, the possibility of lysis-derived or double-membrane subtypes cannot be excluded; both possibilities await examination by in situ cryo-ET imaging and vesicle-subtype isolation experiments.

### 3.2. Regulatory Factors

#### 3.2.1. Decoupled Regulation of EV Yield and Virulence by Quorum Sensing and QSI

**Figure 4:**
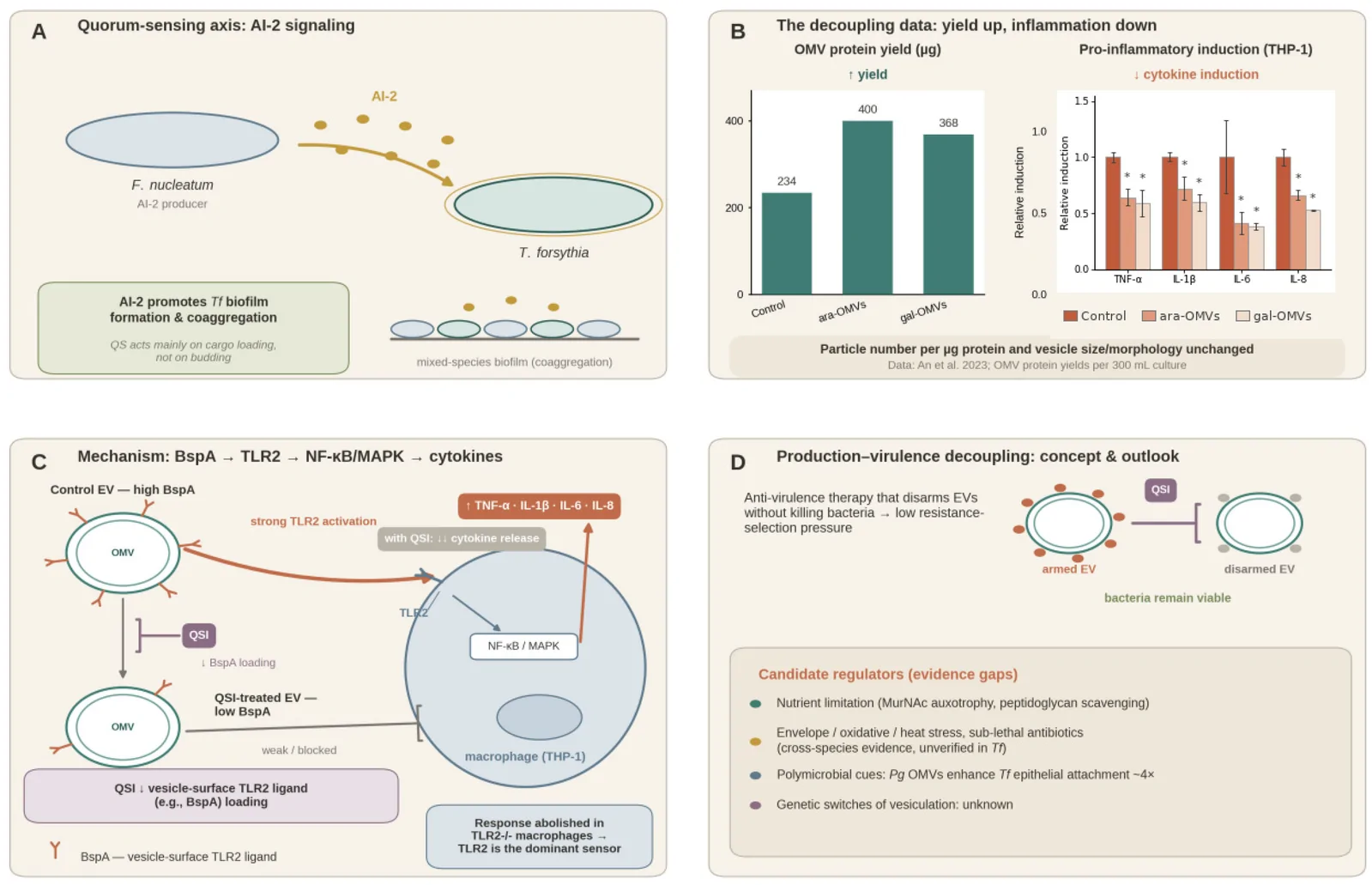
A quorum-sensing inhibitor (QSI) decouples Tf EV yield from virulence. (**A**) AI-2 signals promote Tf biofilm and coaggregation. (**B**) QSI (D-arabinose/D-galactose) raises OMV yield, yet reduces pro-inflammatory activity in THP-1 macrophages. Yield values are single-preparation measurements per 300 mL of culture (no replicate standard deviation reported); cytokine bars show 24 h protein levels (ELISA) normalized to TF OMV controls (mean ± SD of triplicate experiments, redrawn from An et al. [23]); * *p* < 0.05 versus TF OMV control (one-way ANOVA). (**C**) QSI lowers vesicle-surface TLR2-ligand loading, weakening TLR2→NF-κB/MAPK signaling; responses are abolished in TLR2-/- macrophages. (**D**) This yield–virulence decoupling supports anti-virulence therapy that disarms EVs without bactericidal pressure.

Exogenous AI-2 (e.g., quorum-sensing signals derived from *F. nucleatum*) promotes Tf biofilm formation and coaggregation [45]. Particularly critical is the “yield–virulence decoupling” exerted by quorum-sensing inhibitors (QSIs) on EVs (Figure 4): OMV yield in QSI-treated groups did not decrease, but, instead, increased (234 µg protein in controls versus 400 µg for ara-OMVs and 368 µg for gal-OMVs, per 300 mL of culture; particle number per unit protein and size morphology were comparable), yet their capacity to induce TNF-α, IL-1β, IL-6, and IL-8 secretion by THP-1 cells was significantly reduced [23]. Mechanistically, QSIs reduce the loading of vesicle-surface TLR2 ligands (such as BspA [46]), attenuating activation of TLR2 and its downstream NF-κB/MAPK pathways; in TLR2-/- bone-marrow-derived macrophages, the pro-inflammatory activation elicited by Tf OMVs was markedly diminished [23], establishing TLR2 as their principal sensing receptor. This decoupling—increased yield with reduced per-unit virulence—suggests that quorum-sensing regulation targets not budding, per se, but the “quality” of cargo loading, while also providing proof of concept for anti-virulence therapies that disarm without bactericidal pressure and thus carry a low risk of selecting for resistance.

#### 3.2.2. Other Candidate Regulators and Knowledge Gaps

(i) *Nutritional limitation*: the MurNAc auxotrophy of Tf and its dependence on commensal-derived peptidoglycan fragments [39,47] suggest that peptidoglycan homeostasis and membrane–wall linkage status may be coupled to vesicle release [48], but direct experimental evidence is lacking. (ii) *Environmental stress*: general cross-species principles (not Tf-specific evidence) indicate that sublethal antibiotics and oxidative stress can provoke hypervesiculation without cell lysis [49], and heat stress, likewise, upregulates vesicle release [50]; quorum-sensing signals (e.g., PQS) can directly drive vesicle formation [51], and envelope stress, similarly, constitutes a response mechanism regulating vesiculation [49,50]—yet none of these factors has been individually verified in Tf. (iii) *Polymicrobial interactions*: Pg and its OMVs enhance Tf attachment to epithelial cells approximately fourfold via a BspA-independent route, and further amplify BspA-dependent invasion [52], suggesting that vesicle exchange among red-complex members may constitute community-level regulation. It must be stated explicitly that the genetic regulation of Tf EV biogenesis—membrane-curvature-related genes, the coupling switch between T9SS secretion and budding, and the transduction pathways from stress signals to vesiculation phenotypes—is currently supported by no direct evidence, and represents the central gap for future mechanistic research.

## 4. Isolation, Characterization, and Standardization

**Figure 5:**
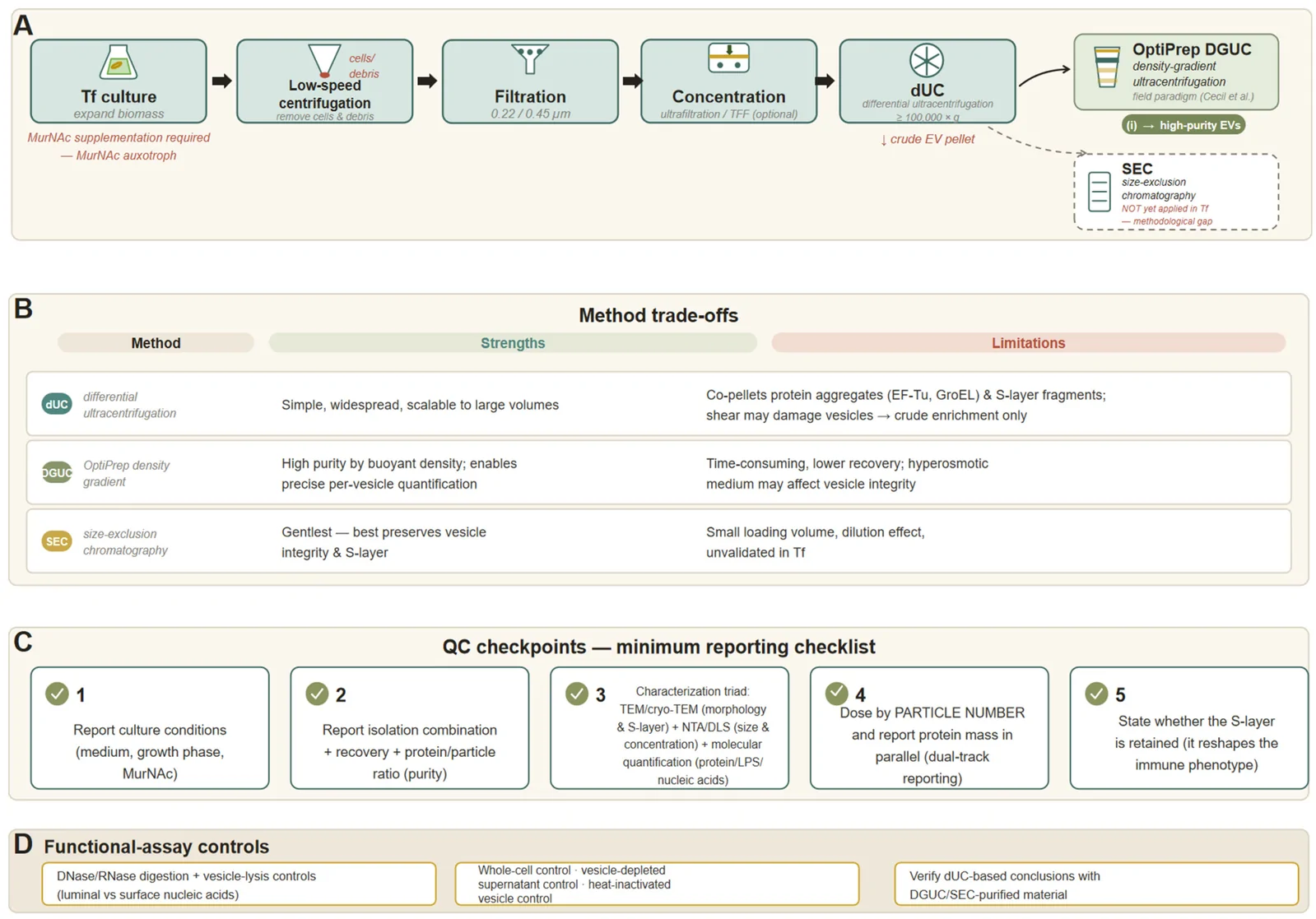
Isolation and quality control of Tf EVs. (**A**) Isolation workflow (dUC ± OptiPrep DGUC [18]; SEC prospective). (**B**) Trade-offs among dUC, DGUC, and SEC. (**C**) Five quality-control checkpoints. (**D**) Recommended functional-assay controls.

Over more than a decade, the methodological framework for *Tannerella forsythia* (Tf) extracellular vesicles (EVs) has evolved from a coarse paradigm of “differential ultracentrifugation plus electron-microscopic confirmation” into a refined one encompassing density-gradient purification, multidimensional physicochemical-fingerprint quantification, and dosing standardized by vesicle number (Table 1). This section reviews the isolation strategies (Figure 5), quantitative characterization, and standardized reporting of Tf EVs, and offers best-practice recommendations benchmarked against the spirit of the MISEV guidelines.

### 4.1. Isolation Strategies

#### 4.1.1. Differential Ultracentrifugation (dUC)

Differential ultracentrifugation (dUC) is the classic workflow of Tf EV research: supernatants are cleared of cells, filtered (0.22/0.45 µm), ultracentrifuged (≥100,000× *g*), and washed [17,23]. It is accessible, simple, and scalable, but co-sedimenting contaminants—free protein aggregates and cytoplasmic proteins (e.g., EF-Tu, GroEL)—are unavoidable [17], confounding cargo attribution and functional specificity. For Tf, the problem is compounded: its OMVs retain an intact S-layer coat, and TfsA/TfsB are abundantly enriched in ultracentrifugation fractions, blurring the distinction between intrinsic cargo and surface-adherent components [17]; high-speed shear may also rupture vesicles or fuse them with contaminants [53]. dUC preparations should therefore be regarded as preliminary enrichments, and dUC-based functional conclusions should be verified with higher-purity preparations [23] (see Section 4.3).

#### 4.1.2. Density-Gradient Ultracentrifugation (DGUC/OptiPrep)

Density-gradient ultracentrifugation (DGUC) separates particles by buoyant density, resolving intact vesicles from denser free-protein and LPS aggregates [54]. Cecil et al. [18] established the field’s paradigm with OptiPrep (iodixanol) purification of Tf OMVs: this high-purity preparation enabled quantification of the entire physicochemical fingerprint, and underpinned the methodological stance of “OptiPrep purification plus dosing by vesicle number rather than protein mass” [18]—protein mass is inflated by co-precipitated contaminants, whereas vesicle number reflects the dose of effector particles (see Section 4.2.3). The costs are longer processing time, lower recovery, and the need to verify the osmotic impact of hyperosmotic media on vesicle integrity.

#### 4.1.3. Size-Exclusion Chromatography (SEC) and Auxiliary Methods

Size-exclusion chromatography (SEC) separates by hydrodynamic size—free proteins enter the resin pores and elute late, whereas vesicles elute first—without centrifugal force or osmotic shock, and is the gentlest on vesicle integrity and S-layer retention [53,55]; ultrafiltration is efficient, but prone to clogging and damage, and polymer precipitation gives high yield but low purity [55]. To date, however, all Tf EV isolation has relied on dUC, in some cases combined with tangential-flow filtration or OptiPrep DGUC, and no SEC-based purification has been reported [17,18,23]. Given the ~20–22 nm S-layer coat of Tf EVs [17,18], a systematic comparison of “dUC + OptiPrep” versus “dUC + SEC” for yield, purity, and S-layer retention is an urgently needed complement.

### 4.2. Quantification and Analytical Characterization

#### 4.2.1. Size and Morphology: TEM/Cryo-ET, DLS/NTA

**Figure 6:**
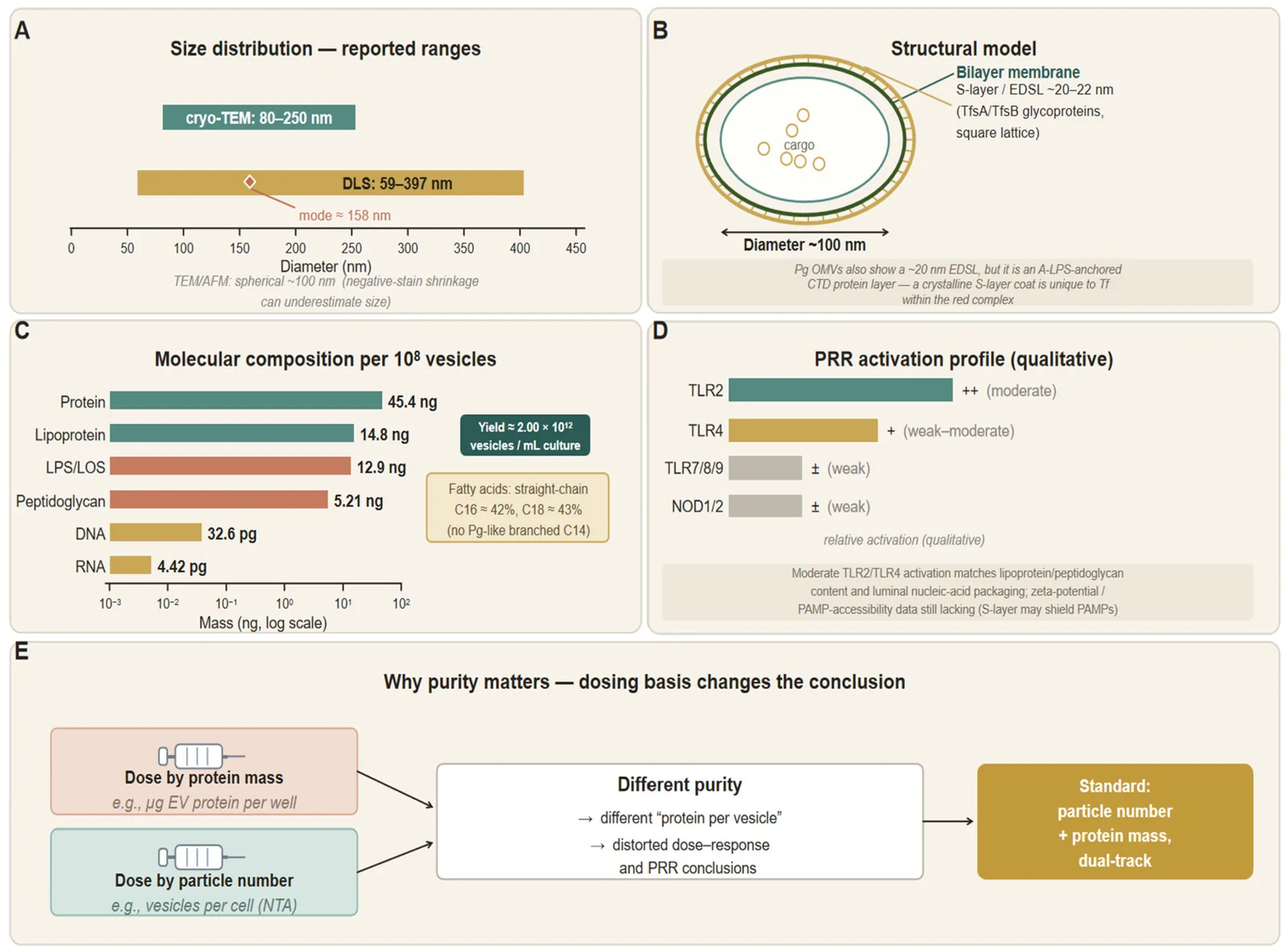
Physicochemical and immunological fingerprint of Tf EVs. (**A**) Size distribution. (**B**) A bilayer coated by a crystalline TfsA/TfsB S-layer—unique within the red complex. (**C**) Per-vesicle molecular composition, yield, and fatty-acid profile. (**D**) PRR profile: moderate TLR2, weak-to-moderate TLR4, weak TLR7/8/9 and NOD1/2. (**E**) Dual-track dosing by particle number and protein mass.

The physicochemical fingerprint of Tf EVs derives from technically complementary methods (Figure 6). Conventional TEM involves fixation and negative staining that can shrink vesicles, so TEM and solution-phase measurements differ systematically [56]; cryo-TEM preserves near-native contours; DLS reports population-averaged hydrodynamic diameters and is sensitive to large particles, whereas NTA tracks particles individually—but neither distinguishes vesicles from protein aggregates, and both require EM cross-validation [56,57]. On this basis, TEM/AFM revealed spherical Tf OMVs of ~100 nm [17]; cryo-TEM gave 80–250 nm and DLS 59–397 nm (mode 158 nm) [18], the DLS mode exceeding the TEM diameter, as expected from staining artifacts. Cryo-TEM and negative-stain TEM further visualized the ~20 nm electron-dense S-layer lattice retained on the vesicle surface [17,18]. Pg vesicles bear a similar ~20 nm layer, but proteomics identifies it as an A-LPS-anchored CTD-protein layer, rather than a crystalline S-layer [58]; a genuine crystalline S-layer armor is unique to Tf within the red complex [17], and may dominate its surface antigenicity and immune recognition.

A dimensional and volumetric audit clarifies what a single Tf EV can physically carry. From the TEM mean diameter of ~100 nm [17], subtracting the ~22 nm S-layer on each side [17] and one outer-membrane bilayer (~7–8 nm) leaves an aqueous lumen of approximately 40–45 nm in diameter—a volume of roughly 3–5 × 10^4^ nm^3^, versus ~5 × 10^5^ nm^3^ for the whole vesicle; the ~25 nm full Gram-negative envelope (inner membrane plus periplasm) would apply only to double-membrane O-IMVs, which have not been demonstrated in Tf [26]. These dimensions translate into a concrete per-vesicle “activity account”: the measured 45.4 ng of protein per 10^8^ vesicles [18] corresponds to ~0.45 fg, i.e., in the order of 10^3^ average-sized protein molecules per vesicle, most of them residing in the membrane and the surface coat, rather than the lumen; the same quantification yields, on average, only ~0.3 kb of DNA and ~80 nt of RNA per vesicle, implying that nucleic-acid cargo is carried by a vesicle subpopulation; and ~50 fmol of miropin per µg of OMV protein [59] corresponds to roughly ten miropin molecules per vesicle. Even allowing for the solvation shells of cargo molecules, a 40–45 nm lumen can accommodate dozens of enzymes, and uptake of only a few vesicles, therefore, delivers a catalytically meaningful, concentrated payload into a single host cell—providing a quantitative explanation for the high effector potency of Tf EVs at low doses [17,21].

#### 4.2.2. Surface Charge and Quantification of Molecular Composition

Cecil et al. [18] provided the most precise per-vesicle quantification to date: per 10^8^ Tf OMVs, protein 45.4 ng, LPS/LOS 12.9 ng, lipoprotein 14.8 ng, peptidoglycan 5.21 ng, DNA 32.6 pg, and RNA 4.42 pg; yield was ~2.00 × 10^12^ vesicles/mL, and fatty acids were dominated by straight-chain C16 (42%) and C18 (43%) [18]. The detected peptidoglycan is noteworthy because Tf is MurNAc-dependent [60]: it may reflect periplasmic packaging during budding or surface adsorption of free fragments [18], and either origin provides a ligand-source explanation—via established OMV–peptidoglycan–NOD signaling [61]—for the weak NOD1/2 activation by Tf OMVs. DNase digestion of Pg OMVs in the same study showed partial digestion resistance, suggesting luminal DNA localization that can be extrapolated to Tf OMVs [18]. Consistently, Tf OMVs activate TLR2/TLR4 moderately and TLR7/8/9 and NOD1/2 only weakly [18]. Whether S-layer glycans mask membrane-surface PAMPs—and thus shape the “moderate TLR2/4 activation” phenotype—remains an unaddressed characterization gap.

#### 4.2.3. Purity Assessment and Standardized Reporting

Purity assessment is the cornerstone of EV dosimetry; the protein/particle ratio, proposed by Webber and Clayton, rapidly discriminates vesicular from non-vesicular protein in a preparation [57]. Cecil et al. [18] further demonstrated that dosing Tf OMVs by vesicle number versus protein mass yields divergent immunological conclusions—medium-derived proteins and debris in crude extracts can alter PRR-activation patterns, and protein-mass standardization flattens inter-species differences—establishing vesicle number as the reporting standard [18]: because “protein per vesicle” varies with purity, mass-based dosing in low-purity preparations overestimates vesicle input and distorts the dose–effect relationships. This principle is crucial for interpreting existing doses: Friedrich et al. reported that Tf OMVs at 0.1–10 µg/mL (by protein) induced stronger inflammatory responses than whole bacteria at 10^7^ cells/mL [17], but mass calibration limits cross-study comparability. Unified dual-track reporting of vesicle number plus protein mass is the realistic bridge [18,57].

Because vesicle-number dosing is the reporting standard advocated above—and applies to Pg and other oral pathogens, likewise—the underlying particle-counting methodology warrants explicit description and critical evaluation. In practice, particle number is obtained by nanoparticle tracking analysis (NTA), which infers size and concentration from the Brownian motion of individually tracked particles [18,56]. Its strengths are speed, single-particle resolution, and direct concentration output; its limitations are equally concrete: (i) NTA cannot discriminate vesicles from protein aggregates, lipoprotein particles, or residual medium components of similar size, so counts from insufficiently purified preparations are systematically inflated [56,57]; (ii) results depend on instrument settings (camera level, and detection threshold) and operator judgment, compromising inter-laboratory comparability, unless settings are reported; and (iii) the lower detection limit (~30–50 nm) undercounts the smallest vesicles. The counts reported for Tf and Pg OMVs were obtained on OptiPrep-purified material, which minimizes—but does not eliminate—these confounders [18]. We therefore recommend that Tf EV studies report NTA settings, dilution, and replicate statistics, and that orthogonal counting methods (e.g., nano-flow cytometry or resistive pulse sensing) be explored for cross-validation as they become established.

### 4.3. Best-Practice Recommendations

Referring to the minimal-reporting spirit of the MISEV guidelines [62,63], and given that reporting standards for bacterial EVs remain ununified [64], we recommend five minimal practices for Tf EV studies: (i) report culture conditions, including medium, growth phase, and MurNAc supplementation [60]; (ii) specify the isolation combination—preferably two-step dUC + OptiPrep DGUC or dUC + SEC—with recovery and purity metrics such as the protein/particle ratio [57]; (iii) complete the characterization triad of TEM/cryo-TEM, NTA/DLS, and molecular quantification of protein/LPS/nucleic acids [17,18]; (iv) report doses by vesicle number, with protein mass provided for cross-study conversion [18]; and (v) state whether the S-layer is retained, as its glycans modulate host immune recognition [17,18].

Functional experiments require purpose-built controls. First, given evidence that vesicle-associated DNA is partially resistant to DNase digestion, suggesting luminal localization [18], nucleic-acid-related functional assays (e.g., TLR9 activation, gene transfer) must include DNase/RNase digestion and vesicle-lysis controls to distinguish surface-adsorbed nucleic acids from luminal cargo. Second, immunological experiments should include at least three classes of controls: whole-bacteria controls (to delimit the relative magnitude of vesicle effects [17]), vesicle-depleted supernatant or free-secreted-protein controls (to exclude contributions of soluble mediators and co-pelleted proteins [18]), and heat-inactivated vesicle controls (to distinguish protein-dependent effects from those of heat-stable components such as LPS [17,18,23]). Third, functional conclusions based on dUC preparations should be verified with DGUC/SEC-purified preparations, as crude and purified preparations can yield different PRR-activation conclusions [18]. Only by institutionalizing these quality-control checkpoints can Tf EV research generate an integrable and verifiable evidence chain.

Strain identity is another underappreciated standardization variable. The studies reviewed here were performed predominantly with the type strain *T. forsythia* ATCC 43037 [17,18,19,20,21,22,23], which we recommend as the reference strain for Tf EV research, so that results remain comparable across laboratories; clinical isolates can differ in surface architecture and virulence, so any deviation from the type strain should be explicitly justified and reported, and systematic comparison of EVs from ATCC 43037 with recent clinical isolates is, itself, a valuable future direction.

Culture technique is a second, equally consequential variable, because the phenotypic traits of EVs are not only strain-specific, but also culture-specific. Tf is a fastidious, strictly anaerobic organism that requires N-acetylmuramic acid (MurNAc) supplementation and grows slowly in broth [39,60]; yields can be improved by co-culture with *F. nucleatum* or by biofilm growth, but co-culture introduces foreign EVs that confound downstream attribution, whereas planktonic broth culture gives cleaner vesicle preparations at lower biomass. Growth phase, medium composition (e.g., serum presence), and stress exposure can all alter EV yield and cargo loading—as illustrated by the quorum-sensing-inhibitor experiments, in which treatment increased OMV yield while reducing virulence-cargo loading [23]. The pros and cons should therefore be weighed explicitly: broth culture favors purity, whereas biofilm and co-culture systems favor physiological relevance; whichever is chosen, culture conditions must be reported in full (see the recommendations above).

## 5. EV Contents

**Figure 7:**
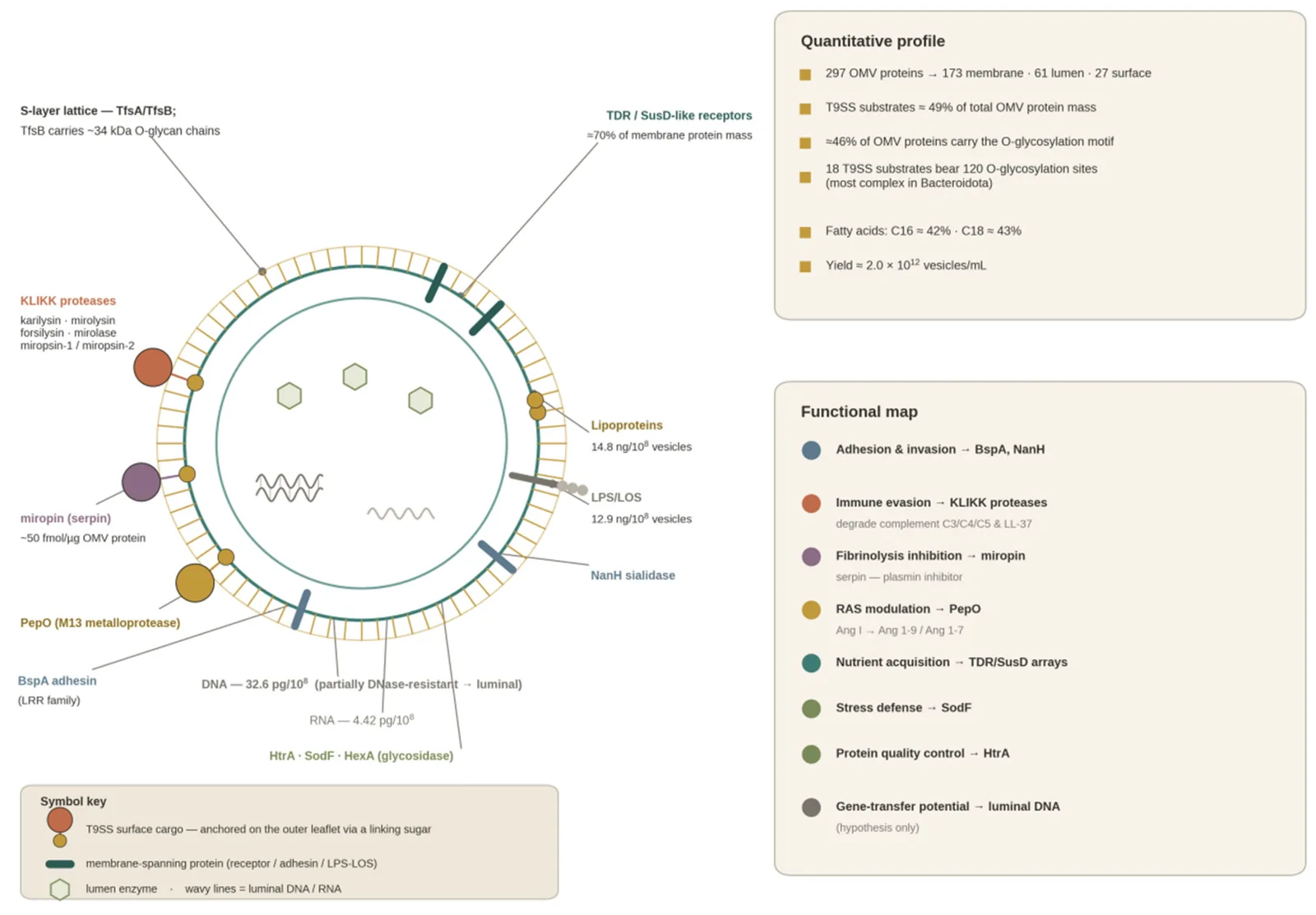
Cargo panorama of Tf EVs. Topology-resolved cross-section: S-layer lattice (TfsA/TfsB), surface-anchored T9SS cargo (KLIKK proteases, miropin, PepO), membrane components (TDR/SusD receptors, lipoproteins, LPS/LOS, BspA, NanH), and luminal cargo (DNA, RNA, HtrA, SodF, HexA), with principal functions indicated.

The pathogenic potential of extracellular vesicles (EVs) is fundamentally determined by their cargo [65,66,67]. Research on the EV cargo of Tf has benefited from the organism’s distinctive secretory biology: as a member of the phylum Bacteroidota, Tf relies heavily on the type IX secretion system (T9SS) to export its surface/secreted proteome [34], a substantial fraction of which is ultimately released via the EV route [19]. This section systematically reviews the compositional evidence for Tf EV cargo (Figure 7) at the protein, lipid, and nucleic-acid levels (Table 2), and explicitly flags the remaining omics gaps.

### 5.1. Proteins

#### 5.1.1. Proteomic Overview: From the First 175-Protein Vesicle Proteome to 297

##### Localization-Resolved OMV Proteins

Tf EV proteomics has advanced across two generations of studies. Friedrich et al. (2015) identified 175 OMV proteins (~35% outer membrane, and ~30% periplasm), confirming BspA, NanH, HtrA, and SodF as cargo, and noted that ~46% carry the O-glycosylation motif [17]. Veith et al. (2015) identified 872 cellular proteins and assigned 297 of them to OMVs (173 membrane, 61 luminal, and 27 surface): T9SS substrates account for ~49% of OMV protein mass, membrane proteins are dominated by TDR/SusD-like proteins (~70%), and KLIKK proteases are conditionally loaded [19]. Sub-vesicular localization refined “what vesicles carry” into a membrane–lumen–surface framework that links cargo topology to potential modes of action [19,68,69], and glycoproteomic analyses add the fact that the 18 T9SS substrates carry 120 O-glycosylation sites (38% of all sites), with occupancy exceeding 99% at most sites [20]. This outer-membrane/surface-dominated pattern is shared with Pg and other Bacteroidota [2,58]; the particularity of Tf lies in the tight coupling of its T9SS cargo to its O-glycosylation apparatus [20]. Tf OMVs additionally carry glycoside hydrolases (NanH, SiaHI, HexA) [17].

#### 5.1.2. Virulence Cargo I: Adhesins and Tissue-Invasion Factors

BspA, a leucine-rich-repeat (LRR)-family adhesin [52], is required for Tf adhesion to and invasion of epithelial cells—anti-BspA serum inhibits invasion by up to 98%, and invasion depends on host F-actin/microtubule rearrangement [52]—and is a confirmed OMV cargo [17]. The sialidase NanH exposes glycan structures for nutritional utilization, mediates epithelial adhesion/invasion [70], and is, likewise, OMV-confirmed [17]. The M13-family metalloendopeptidase PepO is released via OMVs and hydrolyzes angiotensin I, predominantly generating Ang 1–9 (and Ang 1–7 when ACE is inhibited); its unusually wide catalytic cleft renders it refractory to neprilysin- and ACE-inhibitor scaffolds (though metal chelators and phosphoramidon do inhibit it), and Δ*pepO* mutants are significantly attenuated in infection models [24]. HtrA (protease/chaperone) and SodF (superoxide dismutase) are also confirmed OMV cargo [17].

#### 5.1.3. Virulence Cargo II: The KLIKK Protease Family

The KLIKK family—the metalloproteases karilysin, mirolysin, and forsilysin and the serine proteases mirolase, miropsin-1, and miropsin-2—constitutes the most characteristic secreted proteases of Tf [71]: karilysin and mirolysin inhibit all complement-activation pathways (mirolysin also hydrolyzes the antimicrobial peptide LL-37) [72,73,74], whereas the immune-evasion function of mirolase remains unstudied. Miropin, a serpin-family inhibitor, is the first reported prokaryotic proteinaceous plasmin inhibitor (SI = 3.8, kass = 3.3 × 10^5^ M^−1^ s^−1^) [75]; it localizes to the cell surface, is markedly enriched in OMVs (~50 fmol/µg), and reduced bleeding by ~30% in mice [59]. The KLIKK cargo spectra of two independent OMV proteomic studies are not identical (miropsin-2 and forsilysin in [17]; mirolase and karilysin in [19]), and OMV cargo is selectively enriched, relative to cellular fractions [19], indicating conditional, culture-dependent vesicular loading.

#### 5.1.4. S-Layer and O-Glycoprotein Cargo

The Tf surface is covered by an S-layer composed of the TfsA/TfsB glycoproteins [20], a structure that serves simultaneously as a structural component of EVs [17,19] and an immune-recognition component [76,77]. O-glycoproteomic studies have revealed that Tf possesses an exceptionally complex O-glycosylation apparatus: 145 O-glycoproteins, 312 candidate O-glycosylation sites, and at least 13 glycans have been identified, and the O-glycosylation motif has been expanded beyond prior understanding [20], suggesting that its glycosylation machinery can modify a far broader range of substrates than anticipated. The S-layer protein TfsB carries an enormous O-glycan chain of approximately 34 kDa, and exhibits “dual glycosylation” (O-glycan modification coexisting with CTD linking-sugar anchoring) [20]; the TFMS (trifluoromethanesulfonic acid) deglycosylation methodology established in that study, likewise provides a key tool for the identification of cargo glycoproteins. EV-level O-glycoproteomic analyses have experimentally confirmed 120 O-glycosylation sites on the 18 T9SS substrates of Tf OMVs, constituting the most complex O-glycan apparatus in the phylum Bacteroidota. By contrast, the estimate that approximately 46% of OMV proteins contain the O-glycosylation motif [17] derives from sequence-motif analysis, rather than site-level experimental confirmation—the presence of a motif does not prove that a given protein is actually glycosylated—and should therefore be interpreted only as suggesting that O-glycosylation may participate in the directed loading of cargo into vesicles as a candidate sorting signal or stability marker. These glycosylated cargoes not only stabilize vesicle structure, but also directly participate in host immune recognition [17,78].

### 5.2. Lipids

#### 5.2.1. Fatty-Acid and LPS/LOS Composition

Cecil et al. (2016) [18] performed one of the few systematic lipid analyses of Tf OMVs: fatty acids are overwhelmingly dominated by straight-chain C16 (~42%) and C18 (~43%), in contrast to the branched-chain C14 species characteristic of Pg (~58% in Pg) [18]—a difference that, on membrane-biophysics grounds, may enhance bilayer ordering and cargo protection. LOS content was 12.9 ng/10^8^ vesicles and bioactive lipoprotein 14.8 ng/10^8^ vesicles [18], consistent with the moderate TLR2 (and weaker TLR4) activation phenotype, although direct mechanistic verification is still lacking. Of the 98 lipoproteins predicted in the Tf genome [19], only TDR-associated lipoproteins are selectively enriched in OMVs, and direct evidence for lipoprotein-mediated vesicle–host interactions remains lacking [17,19]. In contrast to the systematically dissected penta-/tetra-acylation switching of Pg lipid A [79], the fine structure of Tf LOS/lipid A and its acylation regulation remain entirely unexplored.

#### 5.2.2. Knowledge Gap: Lipidomics and Membrane-Lipid Remodeling

Beyond targeted fatty-acid and LOS quantification, Tf extracellular vesicles still lack systematic untargeted lipidomic analysis—a stark contrast to the relatively deep characterization achieved for OMV protein cargo. In other Gram-negative bacteria, membrane-lipid homeostasis participates in regulating the selection of vesicle-budding sites and the formation of membrane curvature [80]; in the Tf system, however, how membrane-lipid composition participates in vesiculation and membrane-curvature shaping—and whether it participates in anchoring the S-layer at the vesicle surface—remains completely unknown. In addition, whether environmental conditions such as nutrient availability and oxidative stress regulate EV cargo sorting through membrane-lipid remodeling, likewise awaits experimental clarification. The compositional and pattern-recognition-receptor activation data of Cecil et al. suggest that vesicle lipid components both constitute the structural basis of the vesicle membrane and may participate in shaping the host TLR2/TLR4 activation profile [18]. Taken together, these questions should be regarded as priority directions for lipid research on Tf extracellular vesicles.

### 5.3. Nucleic Acids

#### 5.3.1. DNA and RNA Cargo and Its Protective Packaging

Quantitative analysis by Cecil et al. (2016) showed that each 10^8^ Tf OMVs contain 32.6 pg of DNA and 4.42 pg of RNA [18]. DNase-digestion experiments on Pg OMVs in the same study demonstrated that part of the vesicle-associated DNA is digestion-resistant, suggesting luminal localization, rather than surface adsorption; extrapolated to Tf OMVs (not yet independently verified), this implies protective packaging of nucleic-acid cargo in nuclease-rich environments [18]. This resonates with the general mechanism of EV-mediated horizontal transfer of antibiotic-resistance genes [81]: Tf EVs could, theoretically, serve as vehicles for interspecies gene exchange [82]. However, there is currently no direct experimental evidence whatsoever for Tf-EV-mediated horizontal gene transfer—the origin, integrity, and expression fate of vesicular DNA are all unknown, and the notion remains speculative; testing it requires distinguishing DNA topology (plasmid versus genomic fragment) and establishing recipient uptake-and-expression tracing.

#### 5.3.2. Knowledge Gap: A Complete Blank in Small Regulatory RNA (sRNA) Cargo

It must be honestly acknowledged that small regulatory RNA (sRNA) cargo of Tf EVs has not been reported at all to date, constituting one of the most conspicuous omics gaps in the field. For comparison, sRNA research on Pg OMVs has made progress (for example, sRNA45033 has been reported to be delivered via OMVs and to regulate host gene expression [83]—it must be noted that this is Pg evidence, not Tf evidence). Given the increasing prominence of the bacterial sRNA–EV axis in host–pathogen interactions, RNA-seq analysis of Tf EVs should be prioritized as the most urgent omics task.

**Table 2:** Summary of the principal cargo molecules of Tf EVs.

Category	Molecule	Function	References
Adhesin	BspA (LRR adhesin)	Direct EV-level evidence: Tf OMVs adhere via BspA and are internalized by oral epithelial cells [52]. Whole-cell evidence: BspA is required for adhesion/invasion; anti-BspA serum inhibits invasion by 98%, and invasion depends on F-actin/microtubule rearrangement [52].	[17,52]
Glycosidase	NanH (sialidase)	Confirmed OMV cargo [17]; epithelial adhesion/invasion and glycan nutrient-acquisition roles derive from isolated-protein/whole-cell studies, not EV-only activity assays [70].	[17,70]
Glycosidases	Sialidases and glycosidases (lumen-enriched)	Glycan degradation and nutrient acquisition	[19]
Metalloendopeptidase	PepO (M13 family)	Direct EV-associated activity: PepO is released via OMVs and OMV preparations hydrolyze Ang I → Ang 1–7/1–9; Δ*pepO* attenuates this activity; wide catalytic cleft, refractory to classical inhibitors [24].	[24]
Serpin inhibitor	miropin	First prokaryotic serpin and confirmed OMV cargo (~50 fmol/µg OMV protein); plasmin inhibition (SI = 3.8, kass = 3.3 × 10^5^ M^−1^ s^−1^) and ~30% reduction in murine bleeding were demonstrated with isolated/recombinant miropin, whereas the in-vivo activity of EV-delivered miropin remains unverified [59,75].	[59,75]
KLIKK proteases	karilysin, mirolysin, forsilysin, mirolase, miropsin-1, miropsin-2	Conditional OMV cargo [17,19]; degradation of complement and antimicrobial peptides and the resulting immune-evasion activity were demonstrated with isolated KLIKK proteases, rather than EV-only assays [71,72,73,74].	[71,72,73,74]
Proteases/stress defense	HtrA, SodF	Confirmed OMV cargo [17]; protein quality-control and antioxidant functions are inferred from activities of the isolated proteins, not from EV-specific functional assays.	[17]
Lipoproteins	98 lipoproteins predicted from the genome (incl. HmuY, peptidyl-prolyl isomerase)—a whole-cell repertoire, not EV-specific; only TDR-associated lipoproteins are confirmed as selectively enriched in OMVs	Heme uptake/protein folding; lipoprotein 14.8 ng/10^8^ vesicles.	[18,19]
S-layer glycoproteins	TfsA/TfsB	Vesicle structure and immune recognition; TfsB carries ~34-kDa O-glycan chains	[20,29,34]
O-glycoproteome	145 glycoproteins/312 sites/≥13 glycoforms (whole-cell O-glycoproteome, not EV-specific); ~46% of OMV proteins carry the O-glycosylation motif (sequence-motif estimate)	The most complex O-glycan apparatus in Bacteroidota; dual glycosylation; glycosylation linked to vesicle sorting.	[17,20,40]
T9SS substrates	~49% of OMV protein mass; 18 substrates/120 O-glycosylation sites	The core secretory route for cargo sorting and release.	[19,20]
Membrane proteins	TDR/SusD-like proteins (~70% of membrane protein)	Glycan uptake; principal membrane components.	[19]
Lipids	Straight-chain C16 (42%)/C18 (43%) fatty acids; LPS/LOS 12.9 ng/10^8^ vesicles	Membrane structural stability; moderate, TLR2-predominant immune activation.	[18]
Nucleic acids	DNA 32.6 pg, RNA 4.42 pg per 10^8^ vesicles	Luminal protective packaging (DNase-resistant); potential gene-transfer vehicle (hypothesis).	[18]

## 6. Pathogenic Mechanisms

The preceding sections have delineated the biogenesis, physicochemical fingerprint, and cargo composition of *Tannerella forsythia* (Tf) EVs; this section integrates them into a map of disease mechanisms. It must be emphasized that the strength of evidence for Tf EV mechanisms is not uniform: multiple lines of direct EV-level experimental evidence have accumulated for local periodontal inflammation [17,21,22,23], whereas the systemic-disease links largely comprise combinations of “whole-cell/single-virulence-factor evidence plus reasoned extrapolation of EV delivery”. This section presents the evidence in a graded manner: cellular, animal, or clinical data obtained at the EV level are stated in affirmative terms, whereas extrapolations from whole-cell, recombinant-protein, or epidemiological data to the EV context are explicitly flagged within the sentence, so as to avoid overstating the current strength of evidence.

### 6.1. Periodontal Diseases (Periodontitis/Peri-Implantitis/Apical Periodontitis)

**Figure 8:**
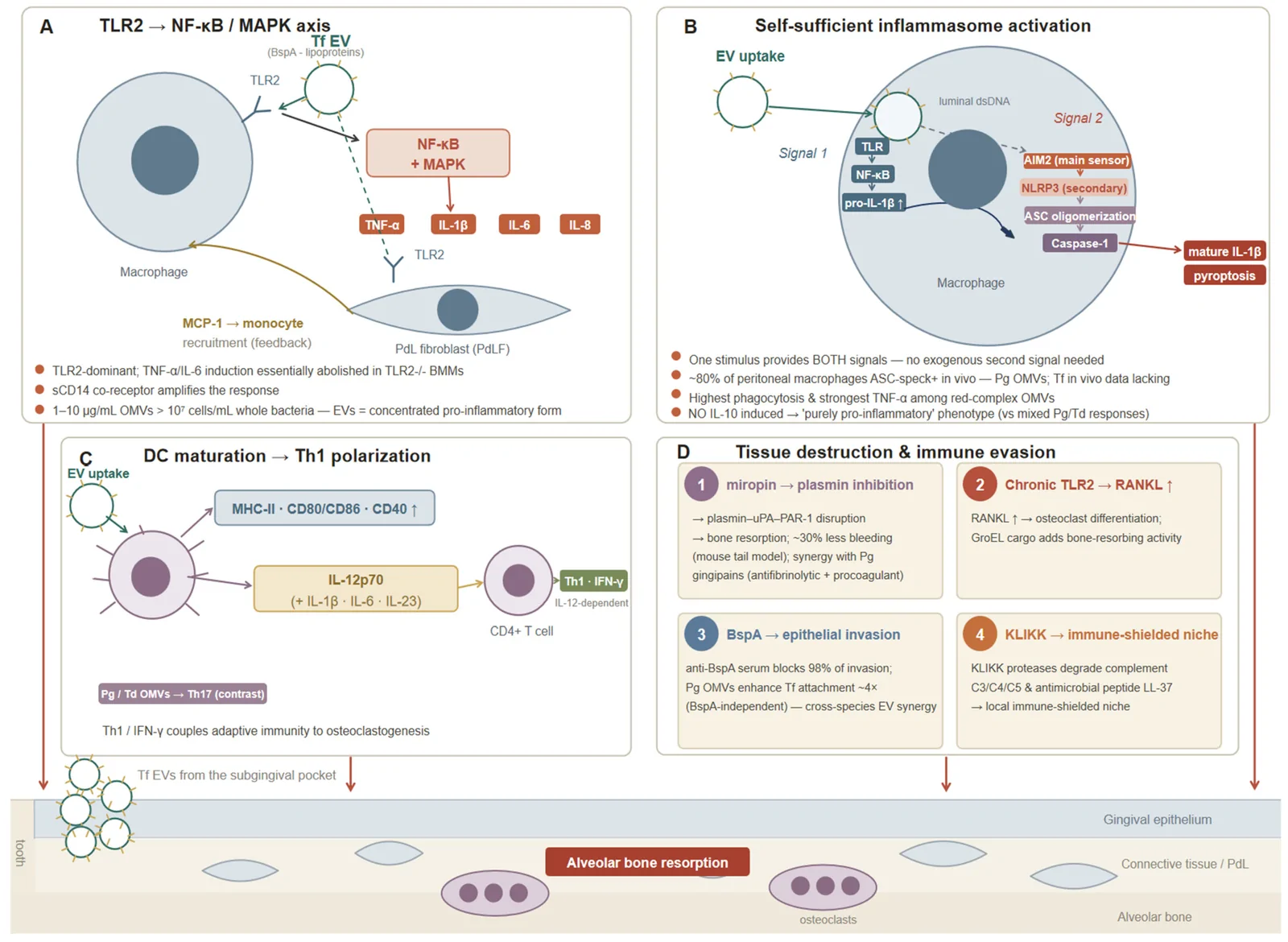
Local pathogenic mechanisms of Tf EVs. (**A**) TLR2-dominant NF-κB/MAPK activation in macrophages and PdL fibroblasts (TNF-α/IL-6 induction essentially abolished in TLR2-/- BMMs). (**B**) Self-sufficient inflammasome activation: one EV provides both signals (TLR priming; AIM2-dominant/NLRP3-secondary activation) → ASC–caspase-1 → IL-1β maturation and pyroptosis (the ~80% in vivo ASC-speck rate refers to Pg OMVs; in vivo data for Tf EVs are lacking); no IL-10—“purely pro-inflammatory”. (**C**) EV-matured DCs drive IL-12-dependent Th1 polarization (vs Th17 for Pg/Td OMVs). (**D**) Tissue-destruction and immune-evasion axes (miropin, RANKL, BspA, KLIKK) converge on alveolar bone resorption.

The periodontal tissues are the “first site” of Tf EV action [17,84]. Current evidence indicates that Tf EVs simultaneously drive three interwoven pathogenic axes (Figure 8)—innate immune activation [23], innate–adaptive immune bridging, and tissue destruction [71,78]—with a potency that in several experiments equals, or even exceeds, that of an equivalent amount of whole bacteria [7,17]. The three axes are highly coupled: the pro-inflammatory milieu of the TLR2 axis creates the conditions for inflammasome priming [85]; IL-1β released by the inflammasome directly enhances antigen-driven expansion and differentiation of CD4 T cells [86]; and DC-polarized Th1 responses converge with osteoclast differentiation on the terminal pathological event of alveolar bone resorption [87].

#### 6.1.1. Innate Immune Activation I: The TLR2→NF-κB/MAPK Axis

The central portal through which Tf EVs activate innate immunity is TLR2, rather than TLR4. An et al. (2023) demonstrated that Tf OMVs activate the NF-κB and MAPK pathways via TLR2, inducing THP-1-derived macrophages to secrete TNF-α, IL-1β, IL-6, and IL-8; these responses were markedly attenuated in TLR2-/- bone-marrow-derived macrophages (BMMs), with TNF-α and IL-6 induction essentially abolished, whereas TLR4 deficiency did not affect the response, identifying TLR2 as the principal receptor mediating these effects in the tested macrophage models [23]. This gene-knockout evidence provides strong causal support for a dominant TLR2-dependent pathway, but does not exclude additional contributions from other PRRs—particularly in other cell types or in vivo—and represents one of the most rigorous experimental designs in the study of Tf EV receptor recognition. This receptor choice is consistent with the molecular composition of the vesicles: they are rich in lipoproteins (14.8 ng/10^8^ vesicles) and T9SS-derived surface proteins, with a moderate LPS/LOS content (12.9 ng/10^8^ vesicles), yielding a PRR profile of “moderate TLR2/4 activation” [18,88]. A recent study further confirmed that Tf OMVs adhere via BspA and are internalized by oral epithelial cells, and that they induce IL-6, IL-8, and MCP-1 in periodontal ligament fibroblasts (PdLFs) at levels exceeding those induced by whole bacteria; Tf and Pg OMVs can additionally create inflammatory conditions for osteoclast differentiation via TLR2 (lipoprotein/LPS-mediated) [6].

Early work had already hinted at the extraordinary pro-inflammatory potency of Tf EVs. Friedrich et al. (2015) reported that Tf OMVs dose-dependently induce TNF-α and IL-8 in U937 monocytes, and that soluble CD14 (sCD14), acting as a PAMP co-receptor, significantly enhances this response—suggesting that the sCD14-positive gingival crevicular fluid environment may further amplify EV pro-inflammatory signaling [17]. In human periodontal ligament fibroblasts (hPdLFs), Tf OMVs induced elevated IL-6, IL-8, and MCP-1, indicating that resident periodontal mesenchymal cells are, likewise, targets, and MCP-1 elevation can recruit monocytes/macrophages to form a positive-feedback loop [17]. Most critically, OMVs at 1–10 µg/mL induced stronger inflammatory responses than whole bacteria at 10^7^ cells/mL, demonstrating that EVs are a highly concentrated form of Tf pro-inflammatory activity—per unit biomass, EVs carry far greater pro-inflammatory potency than whole cells—supporting the view that EVs are the principal executors of long-range, low-dose sustained stimulation by Tf [17]; at the review level, it has likewise been confirmed that the inflammatory responses induced by Tf OMVs via TLR2 equal or exceed those induced by whole bacteria [7]. Taken together, the “TLR2→NF-κB/MAPK→pro-inflammatory cytokine cascade” constitutes the mechanistic axis with the most complete evidence for the periodontal pathogenicity of Tf EVs.

#### 6.1.2. Innate Immune Activation II: Dual-Signal Inflammasome Activation and the “Purely Pro-Inflammatory” Phenotype

The comparative study of OMVs from the three red-complex bacteria by Cecil et al. (2017) provides the deepest inflammasome-mechanism evidence to date, for Tf EVs [21]. Canonical inflammasome activation requires two sequential signals: signal 1 (priming), triggered by TLRs, upregulates pro-IL-1β via NF-κB; signal 2 (activation), triggered by intracellular danger signals, induces sensor oligomerization, which recruits and activates caspase-1 via the adaptor ASC, whereupon caspase-1 cleaves pro-IL-1β to mature IL-1β and triggers pyroptosis. Most bacterial products supply only one of these signals, and require an exogenous second signal (e.g., ATP or nigericin) to complete activation; the uniqueness of Tf OMVs lies in their capacity to provide both signals with a single stimulus—completing priming via TLR pathways while directly triggering inflammasome assembly, without any exogenous second signal [21]. This “self-sufficient” activation capacity means that Tf EVs can independently execute the entire chain from recognition to mature IL-1β release within the periodontal pocket microenvironment, with a pro-inflammatory efficiency far exceeding that of ligand combinations requiring dual stimulation.

The core findings of that study can be summarized in four points: (i) among the three OMVs, Tf OMVs were the most phagocytosed by macrophages and the strongest inducers of TNF-α, and they did not induce the anti-inflammatory cytokine IL-10—displaying a unique “purely pro-inflammatory” phenotype that distinguishes them from the mixed pro-/anti-inflammatory responses of Pg/Td OMVs; (ii) they simultaneously provide both inflammasome signals; (iii) activation is strictly ASC- and caspase-1-dependent, with AIM2 as the dominant sensor and NLRP3 as the secondary one—AIM2 being a cytosolic double-stranded DNA sensor, which suggests that vesicle-delivered DNA cargo may participate in recognition, mechanistically echoing the biochemical evidence of DNase-resistant luminal DNA in Tf OMVs [18,89]; and (iv) in vitro experiments demonstrated that Tf OMVs activate the ASC/caspase-1 inflammasome in a self-sufficient manner. In the same study, intraperitoneal injection of Pg OMVs induced ASC-speck formation in approximately 80% of macrophages in vivo—the gold-standard morphological indicator of inflammasome activation—whereas in vivo activation evidence for Tf OMVs remains lacking; given that an ~80% ASC-speck positivity rate represents a remarkably high activation level in vivo inflammasome research, whether Tf EVs are equally potent inflammasome triggers in circulatory or disseminated settings awaits in vivo validation [21].

The “purely pro-inflammatory” phenotype carries important pathological implications: pathogens such as Pg commonly achieve a balance between immune modulation and immune evasion by co-inducing IL-10, whereas Tf EVs lack this “brake”, and may become a persistently amplifying pro-inflammatory signal source within the periodontal pocket, driving a vicious cycle of chronic inflammation and tissue destruction. From an ecological perspective, the complementarity of the immune phenotypes of the three red-complex EVs (mixed signals for Pg/Td, and a purely pro-inflammatory signal for Tf) may constitute one of the microecological bases by which the red complex sustains chronic inflammation [21].

#### 6.1.3. Innate–Adaptive Immune Bridging: DC Maturation and Distinctive Th1 Polarization

Lim et al. (2022) demonstrated that OMVs of all three red-complex members promote the maturation of bone-marrow-derived dendritic cells (BMDCs), upregulating maturation markers such as MHC-II, CD80, CD86, and CD40 [22]. The general significance of this finding is that nanoscale EVs can deliver bacterial antigens to DCs in “particulate” form, with a presentation efficiency typically exceeding that of soluble antigens [90]; OMVs may thus serve as a bridge connecting local periodontal infection to systemic adaptive immunity. Yet the immune fingerprint of Tf OMVs is distinctive: they induce BMDCs to secrete IL-12p70, IL-1β, IL-6, and IL-23, and they drive CD4^+^ T-cell polarization toward Th1 via an IL-12-dependent pathway; by contrast, Pg and Td OMVs drive Th17 polarization in the same system [22]. IL-12p70 is the decisive cytokine for Th1 polarization, and the IL-12p70/IL-23 combination induced by Tf OMVs (corresponding to Th1 polarization and Th17 maintenance, respectively) demonstrates that its immune fingerprint is absolutely dominated by the Th1 axis [22]. This confers a clear “immunological identity” on Tf EVs: Th1-type responses are closely associated with tissue destruction in active periodontitis, and Th1 effectors such as IFN-γ activate macrophages and promote osteoclastogenesis [87], thereby directly coupling adaptive immunity to bone destruction.

Notably, Tf immune polarization is multilayered: at the S-layer level, its O-glycan terminal trisaccharide drives Th2 polarization and suppresses Th17 via TLR2 [91]; the glycosylation-deficient Δ*wecC* strain is Th17-biased, and exhibits attenuated alveolar bone loss [92], implicating an intact O-glycosylation apparatus in immune modulation and bone destruction [7]. The Th1 polarization at the EV level and the Th2 bias at the S-layer level [91] are not contradictory [22,91]; rather, they may reflect immune routing under different antigenic forms and exposure contexts. Because the S-layer is itself an EV surface component [18,19], how the “Th2 signal of the glyco-epitopes” and the “Th1 signal of the whole cargo” are integrated on the same vesicle is a question worthy of in-depth investigation; this “multilayered immune polarization” phenomenon also cautions against the risk of summarizing the immunological characteristics of Tf in terms of a single polarization pattern.

#### 6.1.4. Molecular Mechanisms of Tissue Destruction and Bone Resorption

Tf EVs may contribute to periodontal tissue damage through at least four molecular pathways, which could converge on osteoclast differentiation and alveolar bone resorption; the strength of evidence differs among these pathways, and direct in-vivo proof of EV-mediated bone destruction in Tf remains limited.

First, inflammatory-signal amplification and protein-processing potential: Tf OMVs cleave pro-IL-1β to mature IL-1β by activating the inflammasome and induce pyroptosis [21], while driving the cascading release of pro-inflammatory cytokines such as TNF-α, IL-6, and IL-8 via the TLR2/NF-κB pathway [17,23]. The protease cargo enriched in OMVs (e.g., PrtH and KLIKK-family members) endows them with the potential to further process host proteins [17,19], suggesting that Tf EVs may reshape the local immune network through the dual mechanism of “inducing inflammation and processing the microenvironment”.

Second, the miropin–plasmin–bone-resorption axis: miropin, the first prokaryotic serpin-family plasmin inhibitor, is enriched in OMVs (approximately 50 fmol/µg OMV protein); by inhibiting plasmin, it interferes with the plasmin–uPA–PAR-1 signaling pathway, and recombinant miropin reduced bleeding by approximately 30% in a mouse tail-bleeding model; the in vivo contribution of EV-delivered miropin has not been established [59]. This antifibrinolytic activity may functionally complement the pro-coagulant activity of Pg gingipains—Tf providing antifibrinolysis and Pg providing pro-coagulation (a mechanistic inference that awaits experimental verification)—suggesting that the pathogenicity of Tf EVs must be understood within a polymicrobial ecological context [59].

Third, BspA-mediated invasion and the polymicrobial OMV synergy network: BspA is required for Tf adhesion to, and invasion of, epithelial cells (anti-BspA serum inhibits invasion by 98%) [52], and has been proteomically confirmed as OMV surface cargo [17,19]. Tf OMVs adhere via BspA and are internalized by oral epithelial cells, providing a direct portal for the intracellular delivery of virulence factors [52]. Of greater ecological significance, Pg OMVs enhance Tf adhesion to epithelial cells approximately fourfold, in a BspA-independent manner, revealing a cross-species EV synergistic virulence network in which the EVs of one bacterium can “pave the way” for the colonization of another [52].

Fourth, KLIKK-protease-mediated immune evasion: KLIKK-family proteases (karilysin, mirolysin, forsilysin, mirolase, miropsin-1, and miropsin-2, among others) degrade key complement components (C3, C4, and C5) and inhibit all three complement-activation pathways—classical, lectin, and alternative [72,74]—and they are conditionally loaded into OMVs [17,19]. In the inflamed gingival crevice, where complement and antimicrobial peptides are enriched, these vesicle-associated proteases could potentially create local “immune-shielded zones”, although this activity has been demonstrated for the isolated enzymes, rather than directly for EV-delivered proteases.

In addition, the potential terminal convergence on osteoclast differentiation and bone resorption: in experimental models, the TLR2-driven chronic inflammatory cascade triggered by Tf OMVs can upregulate RANKL expression, creating conditions favorable for osteoclast differentiation [6,23]. In vivo studies of Pg OMVs have demonstrated more directly that OMVs promote alveolar bone resorption by raising the local periodontal RANKL/OPG ratio [93], suggesting—pending direct validation with Tf EVs in vivo—that “EV recognition → inflammatory cascade → RANKL upregulation → bone destruction” could be a terminal pathogenic pathway shared by red-complex OMVs. Tf OMVs are additionally enriched in proteins known to induce bone resorption, such as GroEL [94], further supporting their potential to contribute to tissue destruction, rather than establishing direct EV-mediated bone loss.

#### 6.1.5. Peri-Implantitis and Apical Periodontitis: Extrapolated Evidence and Gaps

For peri-implantitis, only indirect evidence exists: quorum-sensing inhibitors (QSIs) suppress Tf biofilm formation on titanium surfaces, and although OMV yield increases after QSI treatment, vesicle-surface TLR2-ligand loading decreases and pro-inflammatory activity is significantly attenuated [23]. This finding carries a dual implication: Tf can form biofilms on titanium surfaces and release OMVs, providing a plausible scenario for its participation in peri-implant inflammation [95]; and the “yield–virulence decoupling” phenomenon demonstrates that EV pathogenicity depends on the quality, rather than the quantity, of cargo loading, offering a new rationale for anti-virulence intervention in peri-implantitis—one that targets the attenuation of EV virulence loading, rather than bacterial killing [23,90]. However, there is, as yet, no direct evidence for Tf EVs in peri-implantitis animal models or clinical samples, and multiple steps—the osseointegration interface, host immunity, and the polymicrobial community—still separate the titanium-disc biofilm model from genuine peri-implantitis. For apical periodontitis, no direct Tf EV studies exist, whatsoever; only extrapolation from the anatomy of endodontic–periodontal communication and from the detection of red-complex members in root canals is possible [96]. Both should be honestly labeled as research gaps; for the peri-implantitis direction, the titanium-disc model provides an immediately extensible platform whose barrier to priority validation is relatively low.

### 6.2. Cardiovascular Disease

#### 6.2.1. The BspA–Foam-Cell–Atherosclerosis Axis

The epidemiological association between Tf and cardiovascular disease (CVD) has cohort-level support [8], and Tf DNA has been detected in atherosclerotic plaques [6,97]—demonstrating that Tf genetic material can reach vascular lesions, although such detection does not by itself establish vesicle-mediated transport; as protective carriers of nucleic acids and proteins, EVs are a plausible but unproven candidate for explaining the long-range dissemination of bacterial components [6,18,98]. Mechanistically, BspA induces cytokine production via the CD14/TLR2 pathway, drives the conversion of THP-1 macrophages into foam cells, and aggravates atherosclerosis in ApoE-/- mice [10]; foam-cell formation has likewise been confirmed in Tf-exposure models [6,7]. Given that BspA is a confirmed Tf OMV cargo, and that OMVs are efficiently phagocytosed by macrophages [17,21], the mechanism of “Tf EVs participating in atherosclerosis as circulating BspA-delivery vehicles” remains a hypothesis, rather than an established mechanism: the nanoscale size of EVs could facilitate their evasion of clearance in the circulation and their entry into the vessel wall, where their BspA cargo could be taken up by plaque-resident macrophages. It must be honestly noted, however, that the above evidence derives entirely from the whole-cell or recombinant-protein level; no EV-level animal atherosclerosis experiments exist, and the contribution of Tf EVs to this axis constitutes a well-founded extrapolation, rather than direct evidence.

#### 6.2.2. The PepO–RAS Axis: A Novel Mechanism of Oral–Blood-Pressure Regulation

Waligórska et al. (2025) proposed a novel mechanism: the surface metalloendopeptidase PepO of Tf and Pg is released via OMVs and hydrolyzes angiotensin I (Ang I) to generate Ang 1-7 and Ang 1-9, shifting the renin–angiotensin system (RAS) balance from the pro-inflammatory Ang II axis toward the anti-inflammatory Ang 1-7/1-9 axis [24]. The balance between the pro-inflammatory RAS axis (Ang II mediates vasoconstriction, inflammation, and fibrosis via the AT1 receptor) and the anti-inflammatory axis (Ang 1-7 mediates vasodilation and anti-inflammation via the Mas receptor) is a central regulatory node of blood pressure and cardiovascular homeostasis [99]; the enzymatic activity of PepO amounts to implanting an “RAS diverting valve” in the local oral environment [24]. The crystal structure of TfPepO is unique—an unusually wide catalytic cleft that is refractory to classical M13-family inhibitors; the Δ*pepO* mutant is significantly attenuated in the *Galleria mellonella* model; and, critically, periodontal tissues themselves express a complete set of RAS components, providing an anatomical basis for local “periodontal EV–PepO–RAS” regulation [24]. From this, a “periodontal EV–RAS–blood pressure/cardiovascular” hypothesis can be proposed: Tf EVs reshape the RAS balance at the local and circulatory levels via PepO [99], constituting a previously unrecognized molecular bridge in the periodontitis–CVD association. This hypothesis is bidirectionally testable (the Ang 1-7/1-9-to-Ang II ratio in gingival crevicular fluid or serum; comparison of cardiovascular phenotypes between wild-type- and Δ*pepO*-derived EVs); it should, nonetheless, be stressed that demonstrating the enzymatic activity of the PepO protein does not by itself imply that PepO-bearing EVs exert equivalent effects in vivo, and in vivo validation remains to be completed; at this stage, it should be labeled a mixed evidence level of “direct EV-release evidence with hypothetical cardiovascular outcomes” [24].

### 6.3. Rheumatoid Arthritis (RA)

The epidemiological association between Tf and arthritis has been reviewed [100]. The mechanistic extrapolation runs as follows: Tf OMVs induce BMDCs to secrete IL-12p70 and IL-23—the key polarizing cytokines of the Th1 and Th17 axes, respectively [22]—and Th1/Th17 imbalance is precisely the immunological core of RA synovial inflammation [100]; their potent inflammasome-activating capacity (ASC-dependent, IL-1β maturation) can, likewise, provide a common inflammatory soil for RA-like synovitis [21]. In other words, the two core immunological capabilities displayed by Tf EVs in vitro correspond exactly to the two pillars of RA pathogenesis, lending the extrapolation considerable mechanistic plausibility [21,22]. Three limitations must, nevertheless, be made explicit: (i) Tf lacks a PPAD equivalent of Pg [101] and does not possess the classical mechanism for driving ACPA production, so its route is more likely one of “ACPA-independent inflammatory amplification” [101]; (ii) there is no direct mechanistic evidence whatsoever linking Tf EVs to RA (no synovial models, no EV-level experiments in arthritis animal models); and (iii) the above extrapolation demonstrates only “potential”—the true contribution is entirely unknown, and should be listed as a future direction. The significance of this comparison is that oral-pathogen–RA research has long taken the Pg–PPAD–ACPA axis as its paradigm [101]; the Tf case suggests that a complementary “inflammasome/Th1-axis” route may exist beyond this paradigm, although at the present stage it remains only a hypothesis [21,22].

### 6.4. Respiratory Diseases (The Oral–Lung Axis)

Within the tripartite dissemination framework of metastatic infection/metastatic injury/metastatic inflammation, OMVs are defined as the core vehicles of metastatic injury, delivering virulence factors to distant organs without requiring colonization by viable bacteria [11,98]. This framework is particularly apt for Tf: as a strict anaerobe, Tf has a limited capacity for viable colonization in oxygen-rich lung tissue, yet its EVs can execute virulence delivery in the absence of surviving bacteria, precisely filling the mechanistic gap of “how an anaerobe affects aerobic distant organs” [11]. Evidence supporting the participation of Tf EVs in the oral–lung axis includes the following: the red complex (including Tf) can be detected in both subgingival plaque and tracheal aspirates of patients with acute exacerbations of COPD, suggesting direct oral–airway migration [11,13]; the lung receives the entire cardiac output, and circulating EVs are readily trapped in the pulmonary microvascular bed—this “microvascular trapping” logic implies that even small amounts of Tf EVs entering the circulation would render the lung the principal first-pass trapping organ [11,102]; and the general mechanism of EV-mediated horizontal transfer of antibiotic-resistance genes has clinical implications for airway infections, with the DNase-resistant luminal DNA cargo of Tf EVs theoretically qualifying them as gene-transfer vehicles [11,18]. It should be noted that “airway detection of the red complex” is whole-cell-level evidence; the pulmonary detection and functional validation of Tf EVs themselves remain a blank, and the oral–lung axis falls within the category of “a reasonable framework awaiting direct evidence”.

### 6.5. Head and Neck Squamous-Cell Carcinoma and Gastrointestinal Diseases (Oral–Gut Axis Extrapolation)

It must be honestly noted that there is no direct mechanistic evidence for Tf EVs in head and neck squamous-cell carcinoma (HNSCC), inflammatory bowel disease (IBD), or colorectal cancer (CRC)—neither detection data in tumor tissue or intestinal mucosa, nor EV-level cellular or animal experiments. At present, there are only testable hypotheses proposed by reference to the Fn/Pg OMV oral–gut axis paradigm [14]: (i) the barrier hypothesis—by virtue of their epithelial-internalization capacity and the barrier-disrupting potential of KLIKK proteases [6,7], Tf EVs may weaken intestinal epithelial tight junctions and increase mucosal permeability [103]; (ii) the inflammation–carcinogenesis hypothesis—the “purely pro-inflammatory” macrophage phenotype and TLR2-dominated NF-κB activation could, theoretically, sustain chronic intestinal mucosal inflammation and influence carcinogenic pathways such as Wnt and NF-κB [21,23,103]; and (iii) the microbiota-remodeling hypothesis—swallowed Tf EVs entering the gut may, as bacterial secretions, participate in ecological interactions of the intestinal microbiota [14,104]. These hypotheses can be tested with intestinal epithelial organoids, Caco-2 barrier models, and Wnt reporter assays, but at the present stage they must not be regarded as existing evidence, and should be listed among the highest-priority directions for validation.

### 6.6. Other Systemic Diseases: Alzheimer’s Disease, Adverse Pregnancy Outcomes, and Metabolic Diseases

The epidemiological association of Tf with obesity/type 2 diabetes mellitus (T2DM)-associated periodontitis has been reported [9], and the associations of severe periodontitis with dementia and diabetes have, likewise, been repeatedly emphasized [9,105]. These epidemiological clues point to the necessity of mechanistic exploration: if Tf EVs can indeed carry proteases and pro-inflammatory cargo into the circulation, their potential impact on the cerebral microvasculature and the placental barrier would have clear clinical significance, and could be tested directly with “EV labeling–in vivo tracing” experiments. It must, nevertheless, be made explicit that direct mechanistic evidence for Tf EVs in Alzheimer’s disease (AD) and adverse pregnancy outcomes is completely absent—there are no data whatsoever on entry into the brain or placenta, nor corresponding animal models; what exists are only epidemiological associations and mechanistic analogies extended from Pg OMV research [105,106]. Whether Tf EVs can cross the blood–brain barrier and deliver virulence cargo to distant organs is an entirely open question, and one of the most important evidence gaps delineated in this review.

### 6.7. An Integrated Discussion of the Pathogenic Distinctiveness of Tf EVs

Synthesizing the evidence presented in this section, the pathogenic distinctiveness of Tf EVs relative to the EVs of other oral pathogens such as Pg/Fn can be distilled into five points.

(1) *The structural and immune-recognition properties of the S-layer (EDSL) coat* [29,107]: Tf EVs retain an approximately 20 nm electron-dense S-layer on their surface, which confers a distinctive morphological fingerprint and immune-recognition properties (e.g., Mincle recognition) [7,18]; neither Pg nor Fn EVs possess such a regular surface lattice. The S-layer may simultaneously influence vesicle stability, cellular-uptake routes, and the immune-recognition spectrum, differentiating Tf EVs from other oral-bacterial EVs at the very starting point of pathogenicity—“by whom they are recognized and how they are taken up” [18]. (2) *T9SS-coupled release and cargo sorting*: T9SS substrates account for approximately 49% of OMV protein mass, and cargo loading is selective and even conditional (the KLIKK proteases being a case in point), constituting a coupled “virulence coat–vesicle release” axis that allows the virulence spectrum of Tf EVs to be dynamically adjusted with environmental signals [2,19]. (3) *The most complex O-glycosylation apparatus in Bacteroidota*: 18 T9SS substrates carry 120 O-glycosylation sites, and glycosylation is deeply involved in immune modulation (Th2 bias; attenuated bone loss in Δ*wecC*) [7,20]; the attenuated-bone-loss phenotype of the Δ*wecC* strain directly demonstrates that the glycosylation apparatus is a necessary component of the bone-destructive capacity of Tf [7], while glyco-epitopes (such as the S-layer terminal trisaccharide) are themselves immunomodulatory signals [91], causing the glycosylation layer to assume the dual roles of “structural component” and “immune effector molecule”. (4) *IL-12-dependent Th1 polarization*: in contrast to the Th17 polarization of Pg/Td OMVs, this constitutes the unique adaptive-immunity fingerprint of Tf EVs; the red-complex EVs thus drive two distinct T-cell polarization patterns—Th1 for Tf EVs and Th17 for Pg and Td OMVs, suggesting that the T-cell landscape of chronic periodontitis is the outcome of the superimposed interplay of multiple EV signals [22]. (5) *Signature cargo and synergy networks*: miropin (the plasmin-inhibition–bone-resorption axis) and PepO (RAS remodeling) provide Tf EVs with effector molecules, distinct from those of other oral pathogens [24,59]—the former being the first prokaryotic serpin, the latter suggesting a potential route by which an oral bacterium could influence host blood-pressure regulation, pending in vivo validation; superimposed on the “purely pro-inflammatory” macrophage phenotype and the cross-species OMV synergy network [21,52,108], Tf EVs play the role of both “signal amplifier” and “synergy node” within the red complex. Proteomic comparison further reveals that Pg OMVs are centered on gingipain and adhesin cargo [58], in sharp distinction from the T9SS-substrate/S-layer-dominated pattern of Tf OMVs.

**Figure 9:**
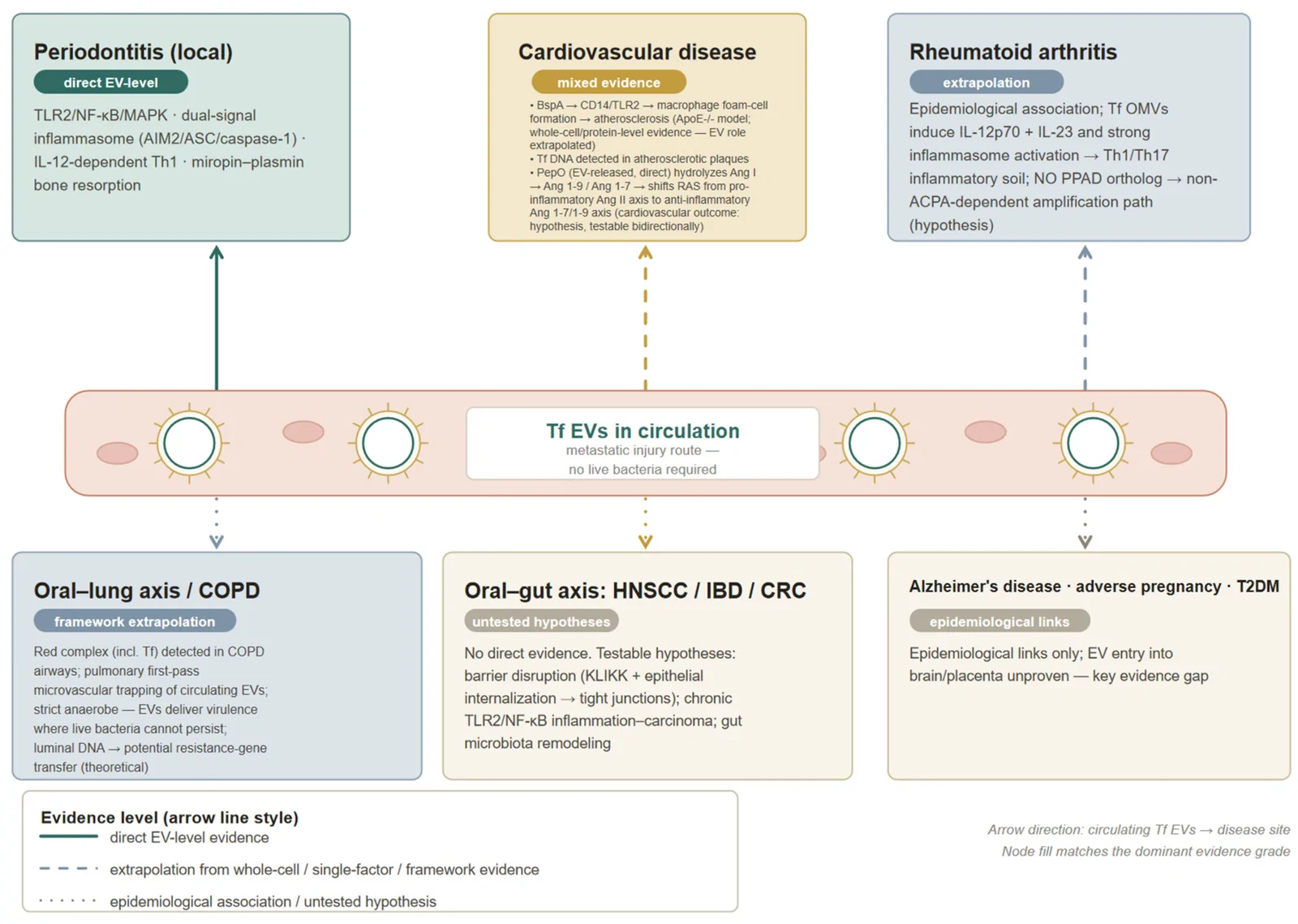
Tf EVs along the oral–systemic disease axes, graded by evidence (solid: direct EV-level; dashed: extrapolation; dotted: epidemiological/hypothesis). Direct EV-level evidence exists only for periodontal disease; cardiovascular and articular links are extrapolative, the oral–lung axis is framework-level, and oral–gut, Alzheimer’s disease, pregnancy, and metabolic links remain untested hypotheses or epidemiological associations.

In summary, Tf EVs are not simply a “scaled-down version” of Pg EVs, but a pathogenic platform with an independent immunological identity and a distinctive cargo logic. Figure 9 maps Tf EVs along the oral–systemic disease axes, and Table 3 summarizes the functions, mechanisms, and evidence levels of Tf EVs across diseases.

A related question deserves explicit comment: given that the EVs of red-complex pathogens share many pathogenic consequences—TLR-driven inflammation, inflammasome activation, and tissue-destructive protease cargo—one may ask whether Tf EVs merit dedicated attention at all, or whether the consortium’s vesicles should instead be considered collectively, when periodontitis and its associated diseases are diagnosed and treated. We agree that, clinically, the polymicrobial EV mixture is the relevant exposure: red-complex EVs act redundantly and synergistically (for example, Pg OMVs enhance Tf attachment to epithelial cells approximately fourfold [52]), and population-level interventions such as intensified oral care or broad anti-virulence strategies need not discriminate among vesicle sources. Nevertheless, three considerations justify a Tf-EV-specific focus. First, the immune phenotypes are not interchangeable: Tf EVs are uniquely “purely pro-inflammatory” (no IL-10 induction), and drive IL-12-dependent Th1 polarization, whereas Pg/Td EVs elicit mixed and Th17-biased responses [21,22]—differences that matter for understanding disease heterogeneity and for designing immunomodulatory interventions. Second, species-specific molecular handles exist: the crystalline S-layer and its conserved O-glycan epitopes [91,109], together with signature cargo such as miropin and PepO, offer a diagnostic specificity that a consortium-level readout cannot provide [59,75]. Third, therapeutic windows may be species-specific: the Pep-7 example—potent against Pg, but ineffective against Tf [110]—demonstrates that anti-EV strategies cannot be extrapolated across the red complex, and must be validated species by species. Thus, while the pathogenic consequences of red-complex EVs converge, their immune recognition, cargo logic, and druggability do not; we argue that Tf EVs warrant recognition as a distinct, diagnostically and therapeutically tractable entity within—rather than apart from—the polymicrobial vesicle community.

## 7. Translational Applications and Clinical Governance

**Figure 10:**
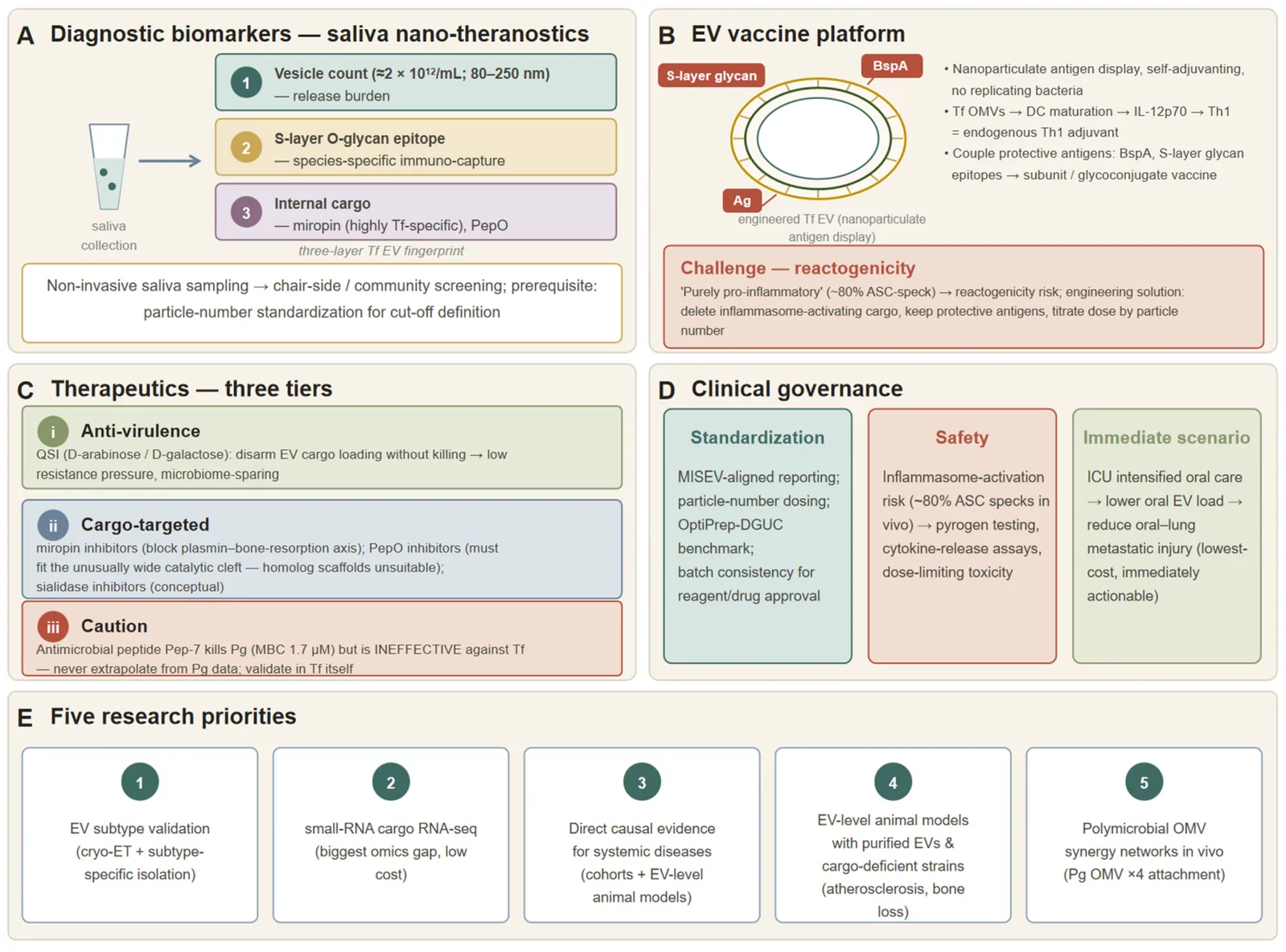
Translational roadmap for Tf EVs. (**A**) Diagnostics via a three-layer molecular fingerprint. (**B**) Self-adjuvanting OMV vaccines with inflammasome-cargo attenuation. (**C**) Therapeutics: QSI anti-virulence and cargo inhibitors. (**D**) MISEV-aligned standardization and safety governance. (**E**) Research priorities.

The preceding sections have delineated the structural and mechanistic basis of *Tannerella forsythia* (Tf) extracellular vesicles (EVs) as a nanoscale virulence-delivery platform. This section translates these insights into three application avenues (Figure 10)—diagnostics, vaccines, and therapeutics—and discusses the standardization, safety, and regulatory-governance thresholds that must be crossed on the path to the clinic.

### 7.1. Translational Applications

#### 7.1.1. Diagnostic Biomarkers and Salivary Nano-Theranostics

Tf EVs possess multiple molecular fingerprints that qualify them as liquid-biopsy biomarkers [111]. First, the vesicles themselves are quantifiable: Tf OMVs are produced at yields as high as 2.00 × 10^12^/mL, with sizes concentrated at 80–250 nm and a reproducible physicochemical fingerprint, providing an analytical target for particle-number-based quantitative detection [18]. Second, the vesicles carry species-specific cargo: miropin, a serpin-family protease inhibitor enriched in OMVs, is a Tf-specific virulence factor (its distribution among other oral commensals remains to be systematically compared) and is, therefore, naturally suited as a differential-diagnostic target [75]; the OMV-released metalloprotease PepO is, likewise, a candidate molecular label [24]. Third, S-layer O-glycan epitopes are conserved within the species, yet are markedly divergent between species [109], supporting highly specific immunocapture assays [91,109]. Together, these three layers constitute a multidimensional fingerprint of “vesicle number + surface glyco-epitopes + internal cargo” [112], which holds promise for developing Tf EVs into liquid-biopsy indicators reflecting periodontitis activity and even systemic inflammatory risk: vesicle number reports the bacterial EV-release burden, the cargo profile indicates the activity of protease-driven tissue destruction, and the glyco-epitopes ensure the species specificity of detection [18,59,91,109]. Regarding the application pathway, the salivary nano-theranostics concept advanced in oral–lung axis research advocates saliva as a non-invasive specimen in which oral-pathogen-derived EVs are simultaneously quantified, molecularly subtyped, and monitored for treatment response, thereby achieving integrated theranostic surveillance; saliva collection is non-invasive and low-cost, and oral EVs are naturally enriched, rendering this approach particularly suitable for community screening and chairside testing [113]. The principal challenge lies in quantitative benchmarking: the methodology established by Cecil et al.—standardizing dose by vesicle number, rather than protein mass—represents foundational work in EV dosimetry [18]; only upon such benchmarks can diagnostic cut-offs such as a “threshold concentration of Tf EVs per milliliter of saliva” be rigorously defined and reproduced across laboratories [18,112].

#### 7.1.2. EV Vaccines and Antigen-Delivery Platforms

Bacterial OMVs concentrate and present multiple native antigens in nanoparticulate form without replicable live bacteria, and the pathogen-associated molecular patterns intrinsic to the vesicle membrane confer self-adjuvanticity, making them an ideal acellular vaccine platform [114]. The immunological properties of Tf OMVs fit this profile: Lim et al. found that OMVs from red-complex members all promote the maturation of bone-marrow-derived dendritic cells (BMDCs), among which Tf OMVs specifically induce IL-12p70 and drive Th1 polarization via an IL-12-dependent pathway—in stark contrast to the Th17-biased profile of Pg and Td OMVs [22]. IL-12p70 is the key cytokine for inducing IFN-γ production and cellular immunity [115], which means that Tf OMVs are not merely antigen carriers, but also possess intrinsic Th1 adjuvant value; coupling them with protective antigens such as BspA and S-layer glyco-epitopes could enable the construction of a Tf subunit vaccine [22]. The concept of glycan-based immunotherapy further suggests that the unique terminal residues of S-layer O-glycans may serve as species-specific candidate epitopes for carbohydrate vaccines or passive immunization [91,116]. The core challenge lies in balancing attenuation against adjuvant activity: Tf OMVs display a “purely pro-inflammatory” phenotype—they fail to induce IL-10 in vitro, and potently activate the inflammasome (in the same study, Pg OMVs induced ASC-speck formation in approximately 80% of macrophages in vivo, whereas in vivo data for Tf remain lacking)—such that their direct use as a vaccine carrier carries a risk of excessive inflammation [21]. A feasible engineering route is therefore to delete strongly inflammasome-activating cargo for “attenuation”, while retaining protective antigens for “efficacy”, and to define the adjuvant-activity window through dose titration by vesicle number [18,21,114].

#### 7.1.3. Nanotherapeutics and Anti-Virulence Therapy

Therapeutic strategies can be organized at three levels. (i) *Anti-virulence therapy*: the quorum-sensing inhibitors (QSIs) D-arabinose and D-galactose suppress AI-2 signaling and titanium-surface biofilm formation in Tf; critically, although OMV yield increases after QSI treatment, TLR2-ligand loading and pro-inflammatory activity decline significantly, and because the bacteria are not killed, resistance-selection pressure is low and commensal communities are minimally disturbed—representing a novel “disarm rather than kill” intervention paradigm [23]. The strength of this mechanism is that the drug targets are virulence-regulatory circuits, rather than growth-essential pathways, so the evolutionary selection pressure exerted is, theoretically, far lower than that of conventional antibiotics, suiting chronic infections such as periodontitis that require long-term management [23,117]. (ii) *Cargo-targeted therapy*: miropin inhibitors could block its “plasmin-inhibition–bone-resorption” axis [59]; PepO inhibitors would need to be designed specifically against the unique wide catalytic cleft of TfPepO, and cannot simply be adapted from the scaffolds of homologous-enzyme inhibitors [24]; and sialidase inhibitors, whose rationale is to strip away the S-layer glycosylation shield and expose antigenic epitopes, remain a conceptual strategy [91]. (iii) *A cautionary negative control*: the antimicrobial peptide Pep-7 specifically kills Pg (MBC 1.7 µM), yet is completely ineffective against Tf [110], demonstrating that membrane properties differ enormously among red-complex members; accordingly, no anti-Tf strategy may be extrapolated from Pg data and must instead be confirmed in Tf itself, lest false-positive expectations arise at the candidate-screening stage [110]. The same logic applies to strategies employing probiotics (e.g., *Lactobacillus* spp.) and plant-derived bioactives (e.g., curcumin) to suppress red-complex OMV-associated virulence genes—this evidence derives mainly from Pg studies, and its applicability to Tf awaits verification, but its low-risk profile still merits exploration as an adjunctive direction [6].

### 7.2. Challenges and Future Directions (Clinical Governance)

Tf EV products face a triple governance bottleneck on the road to the clinic. *Standardization*: EV isolation purity, batch-to-batch consistency, and quantitative units have not yet been unified, and mainstream isolation methods differ markedly in recovery and impurity profiles, rendering cross-study data difficult to compare [64]; the protocol combining vesicle-number quantification with OptiPrep density-gradient purification provides a referenceable benchmark, but its inter-laboratory reproducibility awaits systematic validation [18]. For diagnostic reagents, unified units of measurement are a precondition for establishing reference materials and cut-off values; for therapeutic EV formulations, batch consistency lies at the core of process validation in regulatory review [18]. *Safety*: Tf OMVs potently activate the inflammasome (with ample in vitro evidence; in the same study, Pg OMVs induced ASC-speck formation in approximately 80% of macrophages in vivo, whereas in vivo data for Tf remain lacking) and, among red-complex members, are the most phagocytosed by macrophages and the strongest inducers of TNF-α, while not inducing IL-10 [21]; this implies that EV formulations may carry endotoxin-like systemic inflammatory risk, and for both vaccines and carrier drugs, their inflammasome-activation risk must be incorporated into safety evaluation and regulatory frameworks, including pyrogen testing, cytokine-release assays, and the definition of dose-limiting toxicity [21,118]. *Clinical intervention scenarios*: in high-risk settings such as the ICU, intensified oral care can reduce the oral EV burden and diminish EV-mediated metastatic injury via the oral–lung axis; this is the lowest-cost and most feasible immediate measure, it can be directly integrated into existing ICU oral-care bundles, and it provides a real-world validation scenario for the proposition that “intervening on EV burden improves pulmonary outcomes” [11].

Synthesizing the evidence gaps identified across the preceding sections, we propose five research priorities. (i) *EV subtype validation*: Gram-negative bacteria can produce multiple vesicle subtypes—O-IMVs, EOMVs, and EOIMVs—but whether these subtypes exist in Tf, and how their functions are partitioned, lacks direct evidence; confirmation could be achieved, for example, by cryo-ET and subtype-isolation techniques [119], without excluding emerging alternatives; this is the prerequisite for all subsequent cargo attribution and mechanistic research—if different subtypes selectively load different virulence factors, quantitative conclusions that treat “Tf EVs” as a single analytical object will need to be re-examined. (ii) *Filling the small-RNA cargo gap*: the small regulatory RNA (sRNA) cargo omics of Tf EVs is a complete blank, and constitutes the key shortcoming in understanding their potential for cross-kingdom regulation of host gene expression [120,121]; it could be filled, for instance, by small-RNA-seq and bioinformatic prediction added to existing purified-EV systems—a small investment with a clearly defined output, making it a cost-effective breakthrough in the short term. (iii) *Direct evidence for systemic disease*: the associations of Tf with Alzheimer’s disease, adverse pregnancy outcomes, and intestinal diseases are currently supported only by epidemiology and mechanistic extrapolation; causal experimental evidence at the EV level is urgently needed, with feasible paths including dose–response analyses of blood or salivary Tf EVs against disease phenotypes in clinical cohorts and the reproduction of distal-organ pathology by purified EVs in animal models [98]. (iv) *EV-level animal models*: establishing animal disease models intervened with purified Tf EVs (e.g., atherosclerosis and periodontitis bone-loss models) to validate in vivo the pathological contributions of cargo molecules such as BspA, PepO, and miropin [24,59]; such models directly link “cargo-molecule in vitro activity” to “whole-organism disease phenotype”, and constitute a necessary step for cargo-targeted therapies to enter preclinical development. (v) *In vivo dissection of the polymicrobial OMV synergy network*: Pg OMVs can enhance Tf adhesion to epithelial cells approximately fourfold, in a BspA-independent manner, suggesting a synergistic virulence network among red-complex OMVs; how this network actually operates in mixed biofilms and within the host urgently requires dissection, and may reveal an entirely new intervention window of “breaking synergy”, distinct from single-species targeting [52,108]. The methodologies named above are indicative, rather than prescriptive; the field should remain open to emerging technologies that may supersede them.

In summary, the translational prospects of Tf EVs are clear, while the governance path is long: on the diagnostic side, the leap from molecular fingerprint to standardized reagent must be completed; on the therapeutic side, a fine balance between “disarming” and “safety” must be struck; and filling the five evidence gaps will determine whether this “minute carrier” can ultimately be translated into the clinical reality of “enormous impact.”

## 8. Conclusions and Perspective

This review has systematically integrated the key evidence on *Tannerella forsythia* (Tf) extracellular vesicles (EVs), from which the following core conclusions can be distilled. Tf EVs constitute an S-layer-coated, T9SS-coupled, and highly O-glycosylated nanoscale virulence-delivery platform [2], in which T9SS substrates and TDR/SusD-like proteins are selectively enriched, forming a molecular architecture distinct from that of other oral-pathogen EVs [19]. At the functional level, direct EV-level evidence from cellular models shows that Tf EVs drive local periodontal inflammation through the TLR2/inflammasome/DC–Th1 axis: engaging TLR2 as the recognition portal to activate the NF-κB/MAPK cascade, they simultaneously furnish both signals required for inflammasome activation and exhibit a “purely pro-inflammatory” phenotype, devoid of IL-10 induction [21]; through an IL-12-dependent pathway, they drive Th1 polarization, in stark contrast to the Th17 bias of Pg/Td EVs [22]. At the systemic level, the signature cargo of Tf EVs—BspA, miropin, and PepO—provides plausible mechanistic links between periodontal infection and cardiovascular disease, rheumatoid arthritis, and the oral–lung axis, via the metastatic-injury route [7,11,98,122]; these links, however, rest largely on whole-cell, isolated-protein, and cross-species EV evidence, and direct Tf EV-level demonstration in systemic-disease models remains an outstanding priority. Taken together, the “purely pro-inflammatory” phenotype and the Th1-polarization fingerprint jointly define an immunological identity that distinguishes Tf EVs from Pg/Fn [21,22], laying the conceptual foundation for their recognition as an independent research object and a bona fide intervention target.

Nevertheless, three evidence gaps still constrain the field from achieving mechanistic closure and clinical translation, ranked here by urgency: (i) the gap in causal evidence for systemic disease—the associations of Tf with Alzheimer’s disease, adverse pregnancy outcomes, and intestinal diseases rest solely on epidemiological clues and mechanistic extrapolation, without direct causal evidence at the EV level [106,123]; (ii) the blank in sRNA cargo—the small regulatory RNA (sRNA) cargo omics of Tf EVs remains entirely unreported, to date [121,124]; and (iii) the absence of EV-subtype evidence—whether O-IMV, EOMV, and other vesicle subtypes exist in Tf, and how their functions are partitioned, is unsupported by any direct evidence. Accordingly, this review proposes a four-step roadmap of “subtype isolation → omics dissection → animal modeling → clinical cohorts”: subtype isolation and confirmation—for example, by density-gradient centrifugation combined with cryo-ET or by equivalent emerging techniques; proteomic, glycomic, and small-RNA sequencing of the resolved subtypes to complete the cargo atlas; in vivo causal validation using purified EVs and cargo-deficient strains in animal models of periodontitis bone loss and atherosclerosis; and, ultimately, evaluation of the diagnostic performance of salivary Tf EV biomarkers in clinical cohorts. Along this route, Tf EVs are poised to evolve from “the long-underestimated third pillar of the red complex” into a diagnosable and druggable precision target, opening new avenues for the prevention and management of periodontitis and its systemic comorbidities.

## Figures and Tables

**Table 1:** Summary of methods for the isolation and characterization of Tf EVs.

Method	Principle	Representative References	Advantages	Limitations
Differential ultracentrifugation (dUC)	Stepwise pelleting of vesicles according to differences in sedimentation coefficient	[17,23]	Simple, widely accessible, accommodates large volumes	Co-pellet protein aggregates and S-layer fragments; limited purity
OptiPrep density-gradient ultracentrifugation (DGUC)	Separation in a gradient medium according to buoyant density	[18]	High purity, enabling precise physicochemical quantification	Time-consuming; low recovery; osmotic effects of the medium
Size-exclusion chromatography (SEC)	Gentle separation by hydrodynamic size	Not yet applied in Tf (methodological gap)	Gentle; favorable for preserving the S-layer and vesicle integrity	Small loading volume; dilution effects; unvalidated in Tf
Ultrafiltration/polymer precipitation	Enrichment by membrane retention or polymer co-precipitation	Not yet applied in Tf (methodological gap)	Rapid; high yield	Membrane damage/low purity; unsuitable for fine characterization
TEM/cryo-ET	Electron imaging of morphology and surface structures	[17,18]	Direct visualization of the EDSL/S-layer	Low throughput; dehydration in negative staining can introduce artifacts
DLS/NTA	Light scattering/particle tracking for size and concentration	[18]	Rapid acquisition of size distribution and vesicle counts	Difficulty distinguishing vesicles from protein aggregates
Molecular composition quantification (biochemical assays)	Colorimetric/fluorometric quantification of protein, LPS, nucleic acids, etc.	[18]	Establishes the per-vesicle molecular fingerprint	Requires high-purity preparations; analytes are scattered across assays

**Table 3:** Summary of the functions and mechanisms of Tf EVs across diseases.

Disease	Model	Key Findings	Mechanistic Pathway	Evidence Level	References
Periodontitis (innate immunity)	THP-1 macrophages, TLR2-/-BMMs	Tf OMVs induce TNF-α/IL-1β/IL-6/IL-8; responses markedly attenuated in TLR2-/-	TLR2→NF-κB/MAPK	Direct (EV level)	[23]
Periodontitis (inflammasome)	Macrophages, intraperitoneal injection in mice	Highest phagocytosis, strongest TNF-α induction, no IL-10 induction; self-sufficient inflammasome activation in vitro (the in vivo 80% ASC-speck figure derives from the Pg OMV data of the same study)	Dual inflammasome signals; ASC required; AIM2-dominant/NLRP3-secondary; caspase-1-dependent	Direct (EV level, in vitro; in vivo data from Pg OMVs)	[21]
Periodontitis (adaptive immunity)	BMDC–CD4^+^ T-cell co-culture	Tf OMVs promote DC maturation, induce IL-12p70/IL-23, and drive Th1 polarization (Th17 for Pg/Td)	IL-12-dependent Th1 polarization	Direct (EV level)	[22]
Periodontitis (inflammation induction)	U937, hPdLFs	Dose-dependent TNF-α/IL-8 (enhanced by sCD14); 1–10 µg/mL OMVs outperform 10^7^ cells/mL whole bacteria	Moderate TLR2/4 activation	Direct (EV level)	[17]
Periodontitis (bone resorption)	Osteoclast-differentiation model	Tf/Pg OMVs promote osteoclast differentiation	TLR2 (lipoprotein/LPS-mediated)	Direct (EV level)	[6]
Periodontitis (tissue destruction)	In vivo bleeding model, bone-resorption pathway analysis	Miropin reduces murine bleeding by 30%; interferes with plasmin–uPA–PAR-1 to promote bone resorption; may be functionally complementary to gingipains (inference)	Miropin–plasmin–bone-resorption axis	Direct (in vivo experiments exist for recombinant miropin protein; the in vivo function of EV-delivered miropin has not been directly verified)	[59]
Periodontitis (invasion/synergy)	Epithelial-cell adhesion/invasion models	BspA required for invasion (98% inhibition by antiserum); Pg OMVs enhance Tf adhesion fourfold	BspA-dependent invasion + BspA-independent cross-species synergy	Direct (whole-cell/OMV level)	[6,52]
Periodontitis (immune evasion)	Complement/antimicrobial-peptide degradation assays	KLIKK proteases degrade complement components and antimicrobial peptides; conditionally loaded into OMVs	KLIKK-mediated immune evasion	Direct (cargo level)	[7,19,72,73]
Peri-implantitis	Titanium-disc biofilm + QSI intervention	QSIs suppress titanium-surface biofilms; OMV yield ↑ but pro-inflammatory activity ↓	Reduced TLR2-ligand loading (yield–virulence decoupling)	Indirect (EV level but not a peri-implantitis model)	[23]
Apical periodontitis	—	No direct Tf EV evidence	Extrapolation from endodontic–periodontal communication only	Evidence gap	—
Cardiovascular disease	THP-1 foam cells, ApoE-/- mice	BspA induces foam-cell formation and aggravates atherosclerosis; Tf DNA detected in plaques	BspA–CD14/TLR2–foam-cell axis	Extrapolation (whole-cell/protein level; the EV-vehicle role awaits proof)	[8,10,97]
Cardiovascular/blood-pressure regulation	In vitro enzymology, *Galleria mellonella*	PepO released via OMVs hydrolyzes Ang I → Ang 1-7/1-9; Δ*pepO* attenuated; periodontal tissues express a complete RAS	PepO–RAS anti-inflammatory axis	Direct (EV-release evidence) + hypothesis (cardiovascular outcomes)	[24]
Rheumatoid arthritis	Epidemiology + mechanistic extrapolation	Epidemiological association of Tf with arthritis; Tf OMVs induce IL-12/IL-23 and inflammasome activation; no PPAD ortholog	Th1/Th17 cytokines + inflammasome as a common soil	Extrapolation (no EV evidence in RA models)	[21,22,100]
Respiratory diseases (oral–lung axis)	COPD acute-exacerbation cohort	Red complex detected in airways; OMVs as core vehicles of metastatic injury; pulmonary microvascular trapping	Long-range EV virulence delivery + resistance-gene transfer	Framework evidence (whole-cell-level detection; EVs await proof)	[11]
HNSCC/IBD/CRC	—	No direct evidence; analogy with the oral–gut axis paradigm	Hypotheses: barrier disruption + inflammation/carcinogenesis pathways	Evidence gap (testable hypotheses)	[14]
AD/adverse pregnancy outcomes/T2DM	Epidemiology	Associations of severe periodontitis with dementia and diabetes; association of Tf with obesity/T2DM periodontitis	Unknown	Evidence gap (epidemiological associations only)	[9,105]

## Data Availability

No new data were created or analyzed in this study. Data sharing is not applicable to this article.

## References

[1] Kinane D., Stathopoulou P., Papapanou P. (2017). Periodontal diseases. Nat. Rev. Dis. Primers.

[2] Veith P.D., Glew M.D., Gorasia D.G., Cascales E., Reynolds E.C. (2022). The Type IX Secretion System and Its Role in Bacterial Function and Pathogenesis. J. Dent. Res..

[3] Kebschull M., Papapanou P.N. (2011). Periodontal microbial complexes associated with specific cell and tissue responses. J. Clin. Periodontol..

[4] Socransky S.S., Haffajee A.D. (2002). Dental biofilms: Difficult therapeutic targets. Periodontology.

[5] Socransky S.S., Haffajee A.D. (2005). Periodontal microbial ecology. Periodontology.

[6] Mahendrarajan V., Lazarus H.P.S., Muthukaliannan G.K., Varghese S., Easwaran N. (2025). Membrane vesicles from Red Complex bacteria: Key players in oral pathogenesis, immune disruption, systemic diseases, and therapeutic insights. Front. Oral Health.

[7] Schäffer C., Andrukhov O. (2024). The intriguing strategies of *Tannerella forsythia*’s host interaction. Front. Oral Health.

[8] de Boer S.P., Cheng J.M., Rangé H., Garcia-Garcia H.M., Heo J.H., Akkerhuis K.M., Meilhac O., Cosler G., Pussinen P.J., van Geuns R.J. (2014). Antibodies to periodontal pathogens are associated with coronary plaque remodeling but not with vulnerability or burden. Atherosclerosis.

[9] Lalla E., Papapanou P.N. (2011). Diabetes mellitus and periodontitis: A tale of two common interrelated diseases. Nat. Rev. Endocrinol..

[10] Lee H.R., Jun H.K., Choi B.K. (2014). *Tannerella forsythia* BspA increases the risk factors for atherosclerosis in ApoE(-/-) mice. Oral Dis..

[11] Unlu O., Demirci M., Kantarci A. (2026). Role of Oral-Lung Infection Axis on Respiratory Health. Biomedicines.

[12] Gui M.J., Dashper S.G., Slakeski N., Chen Y.Y., Reynolds E.C. (2016). Spheres of influence: *Porphyromonas gingivalis* outer membrane vesicles. Mol. Oral Microbiol..

[13] Tan L., Wang H., Li C., Pan Y. (2014). 16S rDNA-based metagenomic analysis of dental plaque and lung bacteria in patients with severe acute exacerbations of chronic obstructive pulmonary disease. J. Periodontal Res..

[14] Catalan E.A., Seguel-Fuentes E., Fuentes B., Aranguiz-Varela F., Castillo-Godoy D.P., Rivera-Asin E., Bocaz E., Fuentes J.A., Bravo D., Schinnerling K. (2024). Oral Pathobiont-Derived Outer Membrane Vesicles in the Oral-Gut Axis. Int. J. Mol. Sci..

[15] Zhang Z., Liu D., Liu S., Zhang S., Pan Y. (2021). The Role of *Porphyromonas gingivalis* Outer Membrane Vesicles in Periodontal Disease and Related Systemic Diseases. Front. Cell. Infect. Microbiol..

[16] Zhang X., Wu J., Zhang Z., Liu C., Xie J. (2025). Outer Membrane Vesicles Derived from Oral Bacteria Act as a Dagger in Host Immunity: Insight Tips from Local Diseases to Systemic Effects. Int. J. Biol. Sci..

[17] Friedrich V., Gruber C., Nimeth I., Pabinger S., Sekot G., Posch G., Altmann F., Messner P., Andrukhov O., Schäffer C. (2015). Outer membrane vesicles of *Tannerella forsythia*: Biogenesis, composition, and virulence. Mol. Oral Microbiol..

[18] Cecil J.D., O’Brien-Simpson N.M., Lenzo J.C., Holden J.A., Chen Y.Y., Singleton W., Gause K.T., Yan Y., Caruso F., Reynolds E.C. (2016). Differential Responses of Pattern Recognition Receptors to Outer Membrane Vesicles of Three Periodontal Pathogens. PLoS ONE.

[19] Veith P.D., Chen Y.Y., Chen D., O’Brien-Simpson N.M., Cecil J.D., Holden J.A., Lenzo J.C., Reynolds E.C. (2015). *Tannerella forsythia* Outer Membrane Vesicles Are Enriched with Substrates of the Type IX Secretion System and TonB-Dependent Receptors. J. Proteome Res..

[20] Veith P.D., Scott N.E., Reynolds E.C. (2021). Characterization of the O-Glycoproteome of *Tannerella forsythia*. mSphere.

[21] Cecil J.D., O’Brien-Simpson N.M., Lenzo J.C., Holden J.A., Singleton W., Perez-Gonzalez A., Mansell A., Reynolds E.C. (2017). Outer Membrane Vesicles Prime and Activate Macrophage Inflammasomes and Cytokine Secretion In Vitro and In Vivo. Front. Immunol..

[22] Lim Y., Kim H.Y., An S.J., Choi B.K. (2022). Activation of bone marrow-derived dendritic cells and CD4⁺ T cell differentiation by outer membrane vesicles of periodontal pathogens. J. Oral Microbiol..

[23] An S.J., Ha K.W., Jun H.K., Kim H.Y., Choi B.K. (2023). Reduced proinflammatory activity of outer membrane vesicles of *Tannerella forsythia* treated with quorum sensing inhibitors. Mol. Oral Microbiol..

[24] Waligórska I., Żak K.M., Mikrut N., Budziaszek J., Medviediev V., Bielecka E., Kantyka T., Kozieł J., Thøgersen I.B., Enghild J.J. (2025). Periodontopathogens degrade angiotensin I from the human renin-angiotensin system through surface-attached proteases. Sci. Rep..

[25] Schwechheimer C., Kuehn M.J. (2015). Outer-membrane vesicles from Gram-negative bacteria: Biogenesis and functions. Nat. Rev. Microbiol..

[26] Toyofuku M., Nomura N., Eberl L. (2019). Types and origins of bacterial membrane vesicles. Nat. Rev. Microbiol..

[27] Baeza N., Delgado L., Comas J., Mercade E. (2021). Phage-Mediated Explosive Cell Lysis Induces the Formation of a Different Type of O-IMV in *Shewanella vesiculosa* M7^T^. Front. Microbiol..

[28] Turnbull L., Toyofuku M., Hynen A.L., Kurosawa M., Pessi G., Petty N.K., Osvath S.R., Cárcamo-Oyarce G., Gloag E.S., Shimoni R. (2016). Explosive cell lysis as a mechanism for the biogenesis of bacterial membrane vesicles and biofilms. Nat. Commun..

[29] Sekot G., Posch G., Oh Y.J., Zayni S., Mayer H.F., Pum D., Messner P., Hinterdorfer P., Schäffer C. (2012). Analysis of the cell surface layer ultrastructure of the oral pathogen *Tannerella forsythia*. Arch. Microbiol..

[30] Lasica A.M., Ksiazek M., Madej M., Potempa J. (2017). The Type IX Secretion System (T9SS): Highlights and Recent Insights into Its Structure and Function. Front. Cell. Infect. Microbiol..

[31] Naito M., Tominaga T., Shoji M., Nakayama K. (2019). PGN_0297 is an essential component of the type IX secretion system (T9SS) in *Porphyromonas gingivalis*: Tn-seq analysis for exhaustive identification of T9SS-related genes. Microbiol. Immunol..

[32] Lauber F., Deme J.C., Lea S.M., Berks B.C. (2018). Type 9 secretion system structures reveal a new protein transport mechanism. Nature.

[33] Veith P.D., Shoji M., O’Hair R.A.J., Leeming M.G., Nie S., Glew M.D., Reid G.E., Nakayama K., Reynolds E.C. (2020). Type IX Secretion System Cargo Proteins Are Glycosylated at the C Terminus with a Novel Linking Sugar of the Wbp/Vim Pathway. mBio.

[34] Tomek M.B., Neumann L., Nimeth I., Koerdt A., Andesner P., Messner P., Mach L., Potempa J.S., Schäffer C. (2014). The S-layer proteins of *Tannerella forsythia* are secreted via a type IX secretion system that is decoupled from protein O-glycosylation. Mol. Oral Microbiol..

[35] Shoji M., Sato K., Yukitake H., Naito M., Nakayama K. (2014). Involvement of the Wbp pathway in the biosynthesis of *Porphyromonas gingivalis* lipopolysaccharide with anionic polysaccharide. Sci. Rep..

[36] Veith P.D., Luong C., Tan K.H., Dashper S.G., Reynolds E.C. (2018). Outer Membrane Vesicle Proteome of *Porphyromonas gingivalis* Is Differentially Modulated Relative to the Outer Membrane in Response to Heme Availability. J. Proteome Res..

[37] Orench-Rivera N., Kuehn M.J. (2021). Differential Packaging Into Outer Membrane Vesicles Upon Oxidative Stress Reveals a General Mechanism for Cargo Selectivity. Front. Microbiol..

[38] Glenwright A.J., Pothula K.R., Bhamidimarri S.P., Chorev D.S., Baslé A., Firbank S.J., Zheng H., Robinson C.V., Winterhalter M., Kleinekathöfer U. (2017). Structural basis for nutrient acquisition by dominant members of the human gut microbiota. Nature.

[39] Ruscitto A., Sharma A. (2018). Peptidoglycan synthesis in *Tannerella forsythia*: Scavenging is the modus operandi. Mol. Oral Microbiol..

[40] Tomek M.B., Maresch D., Windwarder M., Friedrich V., Janesch B., Fuchs K., Neumann L., Nimeth I., Zwickl N.F., Dohm J.C. (2018). A General Protein O-Glycosylation Gene Cluster Encodes the Species-Specific Glycan of the Oral Pathogen *Tannerella forsythia*: O-Glycan Biosynthesis and Immunological Implications. Front. Microbiol..

[41] Hoffmanns L., Svedberg D., Mateus A. (2026). Protein O-glycosylation in the *Bacteroidota phylum*. FEBS Open Bio.

[42] Gorasia D.G., Veith P.D., Chen D., Seers C.A., Mitchell H.A., Chen Y.Y., Glew M.D., Dashper S.G., Reynolds E.C. (2015). *Porphyromonas gingivalis* Type IX Secretion Substrates Are Cleaved and Modified by a Sortase-Like Mechanism. PLoS Pathog..

[43] Veith P.D., Shoji M., Scott N.E., Reynolds E.C. (2022). Characterization of the O-Glycoproteome of *Porphyromonas gingivalis*. Microbiol. Spectr..

[44] Ye X., Paul B., Mo J., Reynolds E.C., Ghosal D., Veith P.D. (2024). Ultrastructural and glycoproteomic characterization of *Prevotella intermedia*: Insights into O-glycosylation and outer membrane vesicles. MicrobiologyOpen.

[45] Jang Y.J., Choi Y.J., Lee S.H., Jun H.K., Choi B.K. (2013). Autoinducer 2 of *Fusobacterium nucleatum* as a target molecule to inhibit biofilm formation of periodontopathogens. Arch. Oral Biol..

[46] Onishi S., Honma K., Liang S., Stathopoulou P., Kinane D., Hajishengallis G., Sharma A. (2008). Toll-like receptor 2-mediated interleukin-8 expression in gingival epithelial cells by the *Tannerella forsythia* leucine-rich repeat protein BspA. Infect. Immun..

[47] Ruscitto A., Honma K., Veeramachineni V.M., Nishikawa K., Stafford G.P., Sharma A. (2017). Regulation and Molecular Basis of Environmental Muropeptide Uptake and Utilization in Fastidious Oral Anaerobe *Tannerella forsythia*. Front. Microbiol..

[48] Kulp A., Kuehn M.J. (2010). Biological functions and biogenesis of secreted bacterial outer membrane vesicles. Annu. Rev. Microbiol..

[49] Macdonald I.A., Kuehn M.J. (2013). Stress-induced outer membrane vesicle production by *Pseudomonas aeruginosa*. J. Bacteriol..

[50] McBroom A.J., Kuehn M.J. (2007). Release of outer membrane vesicles by Gram-negative bacteria is a novel envelope stress response. Mol. Microbiol..

[51] Mashburn L.M., Whiteley M. (2005). Membrane vesicles traffic signals and facilitate group activities in a prokaryote. Nature.

[52] Inagaki S., Onishi S., Kuramitsu H.K., Sharma A. (2006). *Porphyromonas gingivalis* vesicles enhance attachment, and the leucine-rich repeat BspA protein is required for invasion of epithelial cells by “*Tannerella forsythia*”. Infect. Immun..

[53] Monguió-Tortajada M., Gálvez-Montón C., Bayes-Genis A., Roura S., Borràs F.E. (2019). Extracellular vesicle isolation methods: Rising impact of size-exclusion chromatography. Cell. Mol. Life Sci. CMLS.

[54] Tulkens J., De Wever O., Hendrix A. (2020). Analyzing bacterial extracellular vesicles in human body fluids by orthogonal biophysical separation and biochemical characterization. Nat. Protoc..

[55] Jia Y., Yu L., Ma T., Xu W., Qian H., Sun Y., Shi H. (2022). Small extracellular vesicles isolation and separation: Current techniques, pending questions and clinical applications. Theranostics.

[56] van der Pol E., Coumans F.A., Grootemaat A.E., Gardiner C., Sargent I.L., Harrison P., Sturk A., van Leeuwen T.G., Nieuwland R. (2014). Particle size distribution of exosomes and microvesicles determined by transmission electron microscopy, flow cytometry, nanoparticle tracking analysis, and resistive pulse sensing. J. Thromb. Haemost. JTH.

[57] Webber J., Clayton A. (2013). How pure are your vesicles?. J. Extracell. Vesicles.

[58] Veith P.D., Chen Y.Y., Gorasia D.G., Chen D., Glew M.D., O’Brien-Simpson N.M., Cecil J.D., Holden J.A., Reynolds E.C. (2014). *Porphyromonas gingivalis* outer membrane vesicles exclusively contain outer membrane and periplasmic proteins and carry a cargo enriched with virulence factors. J. Proteome Res..

[59] Sochaj-Gregorczyk A., Ksiazek M., Waligorska I., Straczek A., Benedyk M., Mizgalska D., Thøgersen I.B., Enghild J.J., Potempa J. (2020). Plasmin inhibition by bacterial serpin: Implications in gum disease. FASEB J. Off. Publ. Fed. Am. Soc. Exp. Biol..

[60] Wyss C. (1989). Dependence of proliferation of *Bacteroides forsythus* on exogenous N-acetylmuramic acid. Infect. Immun..

[61] Kaparakis M., Turnbull L., Carneiro L., Firth S., Coleman H.A., Parkington H.C., Le Bourhis L., Karrar A., Viala J., Mak J. (2010). Bacterial membrane vesicles deliver peptidoglycan to NOD1 in epithelial cells. Cell. Microbiol..

[62] Théry C., Witwer K.W., Aikawa E., Alcaraz M.J., Anderson J.D., Andriantsitohaina R., Antoniou A., Arab T., Archer F., Atkin-Smith G.K. (2018). Minimal information for studies of extracellular vesicles 2018 (MISEV2018): A position statement of the International Society for Extracellular Vesicles and update of the MISEV2014 guidelines. J. Extracell. Vesicles.

[63] Welsh J.A., Goberdhan D.C.I., O’Driscoll L., Buzás E.I., Blenkiron C., Bussolati B., Cai H., Di Vizio D., Driedonks T.A.P., Erdbrügger U. (2024). Minimal information for studies of extracellular vesicles (MISEV2023): From basic to advanced approaches. J. Extracell. Vesicles.

[64] Choi D., Lee E.Y. (2025). Standardizing bacterial extracellular vesicle purification: A call for consensus. J. Microbiol. Biotechnol..

[65] Bomberger J.M., Maceachran D.P., Coutermarsh B.A., Ye S., O’Toole G.A., Stanton B.A. (2009). Long-distance delivery of bacterial virulence factors by *Pseudomonas aeruginosa* outer membrane vesicles. PLoS Pathog..

[66] Kesty N.C., Mason K.M., Reedy M., Miller S.E., Kuehn M.J. (2004). Enterotoxigenic *Escherichia coli* vesicles target toxin delivery into mammalian cells. EMBO J..

[67] Kuehn M.J., Kesty N.C. (2005). Bacterial outer membrane vesicles and the host-pathogen interaction. Genes Dev..

[68] Cvjetkovic A., Jang S.C., Konečná B., Höög J.L., Sihlbom C., Lässer C., Lötvall J. (2016). Detailed Analysis of Protein Topology of Extracellular Vesicles-Evidence of Unconventional Membrane Protein Orientation. Sci. Rep..

[69] Zendrini A., Guerra G., Sagini K., Vagner T., Di Vizio D., Bergese P. (2022). On the surface-to-bulk partition of proteins in extracellular vesicles. Colloids Surf. B Biointerfaces.

[70] Honma K., Mishima E., Sharma A. (2011). Role of *Tannerella forsythia* NanH sialidase in epithelial cell attachment. Infect. Immun..

[71] Ksiazek M., Mizgalska D., Eick S., Thøgersen I.B., Enghild J.J., Potempa J. (2015). KLIKK proteases of *Tannerella forsythia*: Putative virulence factors with a unique domain structure. Front. Microbiol..

[72] Jusko M., Potempa J., Karim A.Y., Ksiazek M., Riesbeck K., Garred P., Eick S., Blom A.M. (2012). A metalloproteinase karilysin present in the majority of *Tannerella forsythia* isolates inhibits all pathways of the complement system. J. Immunol..

[73] Koneru L., Ksiazek M., Waligorska I., Straczek A., Lukasik M., Madej M., Thøgersen I.B., Enghild J.J., Potempa J. (2017). Mirolysin, a LysargiNase from *Tannerella forsythia*, proteolytically inactivates the human cathelicidin, LL-37. Biol. Chem..

[74] Jusko M., Potempa J., Mizgalska D., Bielecka E., Ksiazek M., Riesbeck K., Garred P., Eick S., Blom A.M. (2015). A Metalloproteinase Mirolysin of *Tannerella forsythia* Inhibits All Pathways of the Complement System. J. Immunol..

[75] Ksiazek M., Mizgalska D., Enghild J.J., Scavenius C., Thogersen I.B., Potempa J. (2015). Miropin, a novel bacterial serpin from the periodontopathogen *Tannerella forsythia*, inhibits a broad range of proteases by using different peptide bonds within the reactive center loop. J. Biol. Chem..

[76] Sekot G., Posch G., Messner P., Matejka M., Rausch-Fan X., Andrukhov O., Schäffer C. (2011). Potential of the *Tannerella forsythia* S-layer to delay the immune response. J. Dent. Res..

[77] Settem R.P., Honma K., Nakajima T., Phansopa C., Roy S., Stafford G.P., Sharma A. (2013). A bacterial glycan core linked to surface (S)-layer proteins modulates host immunity through Th17 suppression. Mucosal Immunol..

[78] Chinthamani S., Settem R.P., Honma K., Kay J.G., Sharma A. (2017). Macrophage inducible C-type lectin (Mincle) recognizes glycosylated surface (S)-layer of the periodontal pathogen *Tannerella forsythia*. PLoS ONE.

[79] Reife R.A., Coats S.R., Al-Qutub M., Dixon D.M., Braham P.A., Billharz R.J., Howald W.N., Darveau R.P. (2006). *Porphyromonas gingivalis* lipopolysaccharide lipid A heterogeneity: Differential activities of tetra- and penta-acylated lipid A structures on E-selectin expression and TLR4 recognition. Cell. Microbiol..

[80] Roier S., Zingl F.G., Cakar F., Durakovic S., Kohl P., Eichmann T.O., Klug L., Gadermaier B., Weinzerl K., Prassl R. (2016). A novel mechanism for the biogenesis of outer membrane vesicles in Gram-negative bacteria. Nat. Commun..

[81] Rumbo C., Fernández-Moreira E., Merino M., Poza M., Mendez J.A., Soares N.C., Mosquera A., Chaves F., Bou G. (2011). Horizontal transfer of the OXA-24 carbapenemase gene via outer membrane vesicles: A new mechanism of dissemination of carbapenem resistance genes in *Acinetobacter baumannii*. Antimicrob. Agents Chemother..

[82] Domingues S., Nielsen K.M. (2017). Membrane vesicles and horizontal gene transfer in prokaryotes. Curr. Opin. Microbiol..

[83] Fan R., Zhou Y., Chen X., Zhong X., He F., Peng W., Li L., Wang X., Xu Y. (2023). *Porphyromonas gingivalis* Outer Membrane Vesicles Promote Apoptosis via msRNA-Regulated DNA Methylation in Periodontitis. Microbiol. Spectr..

[84] Lim Y., Kim H.Y., Han D., Choi B.K. (2023). Proteome and immune responses of extracellular vesicles derived from macrophages infected with the periodontal pathogen *Tannerella forsythia*. J. Extracell. Vesicles.

[85] Bauernfeind F.G., Horvath G., Stutz A., Alnemri E.S., MacDonald K., Speert D., Fernandes-Alnemri T., Wu J., Monks B.G., Fitzgerald K.A. (2009). Cutting edge: NF-kappaB activating pattern recognition and cytokine receptors license NLRP3 inflammasome activation by regulating NLRP3 expression. J. Immunol..

[86] Ben-Sasson S.Z., Hu-Li J., Quiel J., Cauchetaux S., Ratner M., Shapira I., Dinarello C.A., Paul W.E. (2009). IL-1 acts directly on CD4 T cells to enhance their antigen-driven expansion and differentiation. Proc. Natl. Acad. Sci. USA.

[87] Teng Y.T., Mahamed D., Singh B. (2005). Gamma interferon positively modulates *Actinobacillus actinomycetemcomitans*-specific RANKL^+^ CD4^+^ Th-cell-mediated alveolar bone destruction in vivo. Infect. Immun..

[88] Teng Y.T., Nguyen H., Gao X., Kong Y.Y., Gorczynski R.M., Singh B., Ellen R.P., Penninger J.M. (2000). Functional human T-cell immunity and osteoprotegerin ligand control alveolar bone destruction in periodontal infection. J. Clin. Investig..

[89] Hornung V., Ablasser A., Charrel-Dennis M., Bauernfeind F., Horvath G., Caffrey D.R., Latz E., Fitzgerald K.A. (2009). AIM2 recognizes cytosolic dsDNA and forms a caspase-1-activating inflammasome with ASC. Nature.

[90] Kaparakis-Liaskos M., Ferrero R.L. (2015). Immune modulation by bacterial outer membrane vesicles. Nat. Rev. Immunol..

[91] Amano A., Chen C., Honma K., Li C., Settem R.P., Sharma A. (2014). Genetic characteristics and pathogenic mechanisms of periodontal pathogens. Adv. Dent. Res..

[92] Settem R.P., Honma K., Sharma A. (2014). Neutrophil mobilization by surface-glycan altered Th17-skewing bacteria mitigates periodontal pathogen persistence and associated alveolar bone loss. PLoS ONE.

[93] Nakamura D., Inagaki Y., Uemura Y., Hiroshima Y., Iwata Y., Kido R., Bando M., Sumitomo T., Yumoto H. (2026). *Porphyromonas gingivalis* outer membrane vesicles promote alveolar bone resorption by increasing the local RANKL/OPG ratio in experimental periodontitis model rats. Sci. Rep..

[94] Jung Y.J., Choi Y.J., An S.J., Lee H.R., Jun H.K., Choi B.K. (2017). *Tannerella forsythia* GroEL induces inflammatory bone resorption and synergizes with interleukin-17. Mol. Oral Microbiol..

[95] Charalampakis G., Belibasakis G.N. (2015). Microbiome of peri-implant infections: Lessons from conventional, molecular and metagenomic analyses. Virulence.

[96] Gomes B.P., Montagner F., Jacinto R.C., Zaia A.A., Ferraz C.C., Souza-Filho F.J. (2007). Polymerase chain reaction of *Porphyromonas gingivalis*, *Treponema denticola*, and *Tannerella forsythia* in primary endodontic infections. J. Endod..

[97] Armingohar Z., Jørgensen J.J., Kristoffersen A.K., Abesha-Belay E., Olsen I. (2014). Bacteria and bacterial DNA in atherosclerotic plaque and aneurysmal wall biopsies from patients with and without periodontitis. J. Oral Microbiol..

[98] Lv C., Shi K., Guo Z., Luo P., Wu Z., Yu P. (2026). *Porphyromonas gingivalis*-derived outer membrane vesicles travel from oral to distant organs via blood circulation in a time-dependent manner. BMC Oral Health.

[99] Santos R.A.S., Sampaio W.O., Alzamora A.C., Motta-Santos D., Alenina N., Bader M., Campagnole-Santos M.J. (2018). The ACE2/Angiotensin-(1-7)/MAS Axis of the Renin-Angiotensin System: Focus on Angiotensin-(1-7). Physiol. Rev..

[100] McInnes I.B., Schett G. (2011). The pathogenesis of rheumatoid arthritis. N. Engl. J. Med..

[101] Wegner N., Wait R., Sroka A., Eick S., Nguyen K.A., Lundberg K., Kinloch A., Culshaw S., Potempa J., Venables P.J. (2010). Peptidylarginine deiminase from *Porphyromonas gingivalis* citrullinates human fibrinogen and α-enolase: Implications for autoimmunity in rheumatoid arthritis. Arthritis Rheum..

[102] Fischer U.M., Harting M.T., Jimenez F., Monzon-Posadas W.O., Xue H., Savitz S.I., Laine G.A., Cox C.S. (2009). Pulmonary passage is a major obstacle for intravenous stem cell delivery: The pulmonary first-pass effect. Stem Cells Dev..

[103] Rubinstein M.R., Wang X., Liu W., Hao Y., Cai G., Han Y.W. (2013). *Fusobacterium nucleatum* promotes colorectal carcinogenesis by modulating E-cadherin/β-catenin signaling via its FadA adhesin. Cell Host Microbe.

[104] Wang X., Jia Y., Wen L., Mu W., Wu X., Liu T., Liu X., Fang J., Luan Y., Chen P. (2021). *Porphyromonas gingivalis* Promotes Colorectal Carcinoma by Activating the Hematopoietic NLRP3 Inflammasome. Cancer Res..

[105] Asher S., Stephen R., Mäntylä P., Suominen A.L., Solomon A. (2022). Periodontal health, cognitive decline, and dementia: A systematic review and meta-analysis of longitudinal studies. J. Am. Geriatr. Soc..

[106] Pritchard A.B., Fabian Z., Lawrence C.L., Morton G., Crean S., Alder J.E. (2022). An Investigation into the Effects of Outer Membrane Vesicles and Lipopolysaccharide of *Porphyromonas gingivalis* on Blood-Brain Barrier Integrity, Permeability, and Disruption of Scaffolding Proteins in a Human in vitro Model. J. Alzheimer’s Dis. JAD.

[107] Lee S.W., Sabet M., Um H.S., Yang J., Kim H.C., Zhu W. (2006). Identification and characterization of the genes encoding a unique surface (S-) layer of *Tannerella forsythia*. Gene.

[108] Kendlbacher F.L., Afzal M., Andrukhov O., Schäffer C. (2026). *Tannerella forsythia*–*Porphyromonas gingivalis* polymicrobial biofilm synergy boosts host inflammation and remodels the *T. forsythia* transcriptome. npj Biofilms Microbiomes.

[109] Posch G., Pabst M., Brecker L., Altmann F., Messner P., Schäffer C. (2011). Characterization and scope of S-layer protein O-glycosylation in *Tannerella forsythia*. J. Biol. Chem..

[110] Suwandecha T., Srichana T., Balekar N., Nakpheng T., Pangsomboon K. (2015). Novel antimicrobial peptide specifically active against *Porphyromonas gingivalis*. Arch. Microbiol..

[111] Tamura T., Yoshioka Y., Sakamoto S., Ichikawa T., Ochiya T. (2021). Extracellular vesicles as a promising biomarker resource in liquid biopsy for cancer. Extracell. Vesicles Circ. Nucleic Acids.

[112] Liang Y., Lehrich B.M., Zheng S., Lu M. (2021). Emerging methods in biomarker identification for extracellular vesicle-based liquid biopsy. J. Extracell. Vesicles.

[113] Han P., Bartold P.M., Ivanovski S. (2022). The emerging role of small extracellular vesicles in saliva and gingival crevicular fluid as diagnostics for periodontitis. J. Periodontal Res..

[114] Garling A., Auvray F., Epardaud M., Oswald É., Branchu P. (2025). Outer Membrane Vesicles as a Versatile Platform for Vaccine Development: Engineering Strategies, Applications and Challenges. J. Extracell. Vesicles.

[115] Trinchieri G. (2003). Interleukin-12 and the regulation of innate resistance and adaptive immunity. Nat. Rev. Immunol..

[116] Terra V.S., Mills D.C., Yates L.E., Abouelhadid S., Cuccui J., Wren B.W. (2012). Recent developments in bacterial protein glycan coupling technology and glycoconjugate vaccine design. J. Med. Microbiol..

[117] Rasko D.A., Sperandio V. (2010). Anti-virulence strategies to combat bacteria-mediated disease. Nat. Rev. Drug Discov..

[118] Vipond C., Sutherland J., Nordgren K., Kemp G., Heath A., Care R., Studholme L. (2019). Development and validation of a monocyte activation test for the control/safety testing of an OMV-based meningococcal B vaccine. Vaccine.

[119] Pérez-Cruz C., Delgado L., López-Iglesias C., Mercade E. (2015). Outer-inner membrane vesicles naturally secreted by gram-negative pathogenic bacteria. PLoS ONE.

[120] Lécrivain A.L., Beckmann B.M. (2020). Bacterial RNA in extracellular vesicles: A new regulator of host-pathogen interactions?. Biochim. Biophys. Acta Gene Regul. Mech..

[121] Dauros-Singorenko P., Blenkiron C., Phillips A., Swift S. (2018). The functional RNA cargo of bacterial membrane vesicles. FEMS Microbiol. Lett..

[122] Hayashi C., Gudino C.V., Gibson F.C., Genco C.A. (2010). Review: Pathogen-induced inflammation at sites distant from oral infection: Lessons from *Porphyromonas gingivalis*. Mol. Oral Microbiol..

[123] Lara B., Loureiro I., Gliosca L., Castagnola L., Merech F., Gallino L., Calo G., Sassot M., Ramhorst R., Vota D. (2023). *Porphyromonas gingivalis* outer membrane vesicles shape trophoblast cell metabolism impairing functions associated to adverse pregnancy outcome. J. Cell. Physiol..

[124] Ha J.Y., Kim S.M., Choi S.Y., Park C., Park S., Hong S.H., Jang I.S., Lee Y., Lee H.J. (2026). Intranasal Delivery of Bacterial Extracellular Vesicles Enables RNA Cargo Entry Into the Brain. J. Extracell. Vesicles.

